# IL-26 mediates epidermal growth factor receptor-tyrosine kinase inhibitor resistance through endoplasmic reticulum stress signaling pathway in triple-negative breast cancer cells

**DOI:** 10.1038/s41419-021-03787-5

**Published:** 2021-05-21

**Authors:** Takumi Itoh, Ryo Hatano, Yoshiya Horimoto, Taketo Yamada, Dan Song, Haruna Otsuka, Yuki Shirakawa, Shuji Mastuoka, Noriaki Iwao, Thomas M. Aune, Nam H. Dang, Yutaro Kaneko, Ko Okumura, Chikao Morimoto, Kei Ohnuma

**Affiliations:** 1 Department of Therapy Development and Innovation for Immune Disorders and Cancers, Graduate School of Medicine, Juntendo University, 2-1-1, Hongo, Bunkyo-ku, Tokyo 113-8421 Japan; 2 Atopy (Allergy) Research Center, Graduate School of Medicine, Juntendo University, 2-1-1, Hongo, Bunkyo-ku, Tokyo 113-8421 Japan; 3 Department of Breast Oncology, School of Medicine, Juntendo University, 2-1-1, Hongo, Bunkyo-ku, Tokyo 113-8421 Japan; 4 Department of Pathology, Saitama Medical University, 38 Morohongo, Moroyama-machi, Iruma-gun, Saitama 350-0495 Japan; 5 Department of Pathology, Keio University School of Medicine, 35 Shinanomachi, Shinjuku-ku, Tokyo 160-8582 Japan; 6 Department of Immunological Diagnosis, Graduate School of Medicine, Juntendo University, 2-1-1, Hongo, Bunkyo-ku, Tokyo 113-8421 Japan; 7 Department of Hematology, Juntendo University Shizuoka Hospital, 1129 Nagaoka, Izunokuni, Shizuoka 410-2295 Japan; 8 Department of Medicine, Vanderbilt University School of Medicine, Vanderbilt University Medical Center, Nashville, TN 37232 USA; 9 Division of Hematology/Oncology, University of Florida, 1600 SW Archer Road-Box 100278, Room MSB M410A, Gainesville, FL 32610 USA; 10 Y’s AC Co., Ltd., 2-6-8 Kudanminami, Chiyoda-ku, Tokyo 102-0074 Japan

**Keywords:** Stress signalling, Cell death and immune response, Breast cancer

## Abstract

Triple-negative breast cancer (TNBC) has a poor prognosis compared to other breast cancer subtypes. Although epidermal growth factor receptor (EGFR) is overexpressed in TNBC, clinical trials with EGFR inhibitors including tyrosine kinase inhibitors (EGFR-TKI) in TNBC have heretofore been unsuccessful. To develop effective EGFR-targeted therapy for TNBC, the precise mechanisms of EGFR-TKI resistance in TNBC need to be elucidated. In this study, to understand the molecular mechanisms involved in the differences in EGFR-TKI efficacy on TNBC between human and mouse, we focused on the effect of IL-26, which is absent in mice. In vitro analysis showed that IL-26 activated AKT and JNK signaling of bypass pathway of EGFR-TKI in both murine and human TNBC cells. We next investigated the mechanisms involved in IL-26-mediated EGFR-TKI resistance in TNBC. We identified EphA3 as a novel functional receptor for IL-26 in TNBC. IL-26 induced dephosphorylation and downmodulation of EphA3 in TNBC, which resulted in increased phosphorylation of AKT and JNK against EGFR-TKI-induced endoplasmic reticulum (ER) stress, leading to tumor growth. Meanwhile, the blockade of IL-26 overcame EGFR-TKI resistance in TNBC. Since the gene encoding IL-26 is absent in mice, we utilized human *IL-26* transgenic (hIL-26Tg) mice as a tumor-bearing murine model to characterize the role of IL-26 in the differential effect of EGFR-TKI in human and mice and to confirm our in vitro findings. Our findings indicate that IL-26 activates the bypass pathway of EGFR-TKI, while blockade of IL-26 overcomes EGFR-TKI resistance in TNBC via enhancement of ER stress signaling. Our work provides novel insights into the mechanisms of EGFR-TKI resistance in TNBC via interaction of IL-26 with its newly identified receptor EphA3, while also suggesting IL-26 as a possible therapeutic target in TNBC.

## Introduction

Accounting for 15–20% of breast cancer, triple-negative breast cancer (TNBC) subtype lacks estrogen receptor, progesterone receptor (PgR), and human epidermal growth factor receptor 2 (HER2) expression and has a poor prognosis^1–4^. To date, no effective molecular target has been identified in TNBC^5^. Epidermal growth factor receptor (EGFR) signaling pathway is activated in various neoplasms, and EGFR-tyrosine kinase inhibitors (EGFR-TKIs) are used as targeted therapy in these cancers^6^. While EGFR is highly expressed in most TNBC and preclinical models showed a significant effect of EGFR-TKIs on TNBC^7,8^, results from clinical studies of EGFR-TKIs for TNBC have been disappointing^9,10^. The molecular mechanisms involved in the differences in efficacy of EGFR-TKI on TNBC between human and mouse model should be elucidated to overcome EGFR-TKI resistance in TNBC.

Human IL-26, mainly produced by Th1, Th17, or NK cells, belongs to the IL-10 cytokine family and regulates T cells, monocytes, NK cells, synoviocytes, fibroblasts, and bacterial pathogens in the inflammatory lesions^11–15^. We previously reported that IL-26 secreted by CD4 + T cells activates fibroblasts for collagen production via its functional receptor IL-20RA/IL-10RB, and that IL-26 has an important role in lung fibrosis of chronic graft-versus-host disease^13^. IL-26 also directly acts on endothelial cells to induce angiogenesis, equivalent to the effect of vascular endothelial growth factor (VEGF) at inflammatory sites^14^, with a potential role in angiogenesis in the tumor microenvironment (TME) and malignant progression. In addition, IL-26 has a role in the development of other cancers including gastric cancer and hepatocellular carcinoma^16–18^, while little is known about its function in breast cancer. Importantly, human IL-26 is conserved in several vertebrate species but not found in rodents including mice and rats^11,19^. The lack of IL-26 in appropriate murine models has impeded research to understand cross-species differences in EGFR-TKI susceptibility or resistance in TNBC. To address this important issue, we now expand on our previous findings and define the molecular mechanisms involved in IL-26-mediated EGFR-TKI resistance in TNBC, and establish that IL-26 is an appropriate therapeutic target in TNBC.

Various factors such as nutrient deprivation, hypoxia, and loss of calcium homeostasis provoke endoplasmic reticulum (ER) stress^20,21^. When unfolded/misfolded proteins accumulate within ER above a critical threshold, the ER stress response is induced to restore homeostasis^20,22^. The ER stress response is mainly initiated via three signal transducers located in the ER known as protein kinase RNA-like ER kinase (PERK), IRE1α, and activating transcription factor (ATF)-6, and is regulated by BCL2 family proteins^23–26^. However, when the ER stress signals are too strong to restore homeostasis, those signals promote apoptosis and cytotoxicity^24–26^. The PERK‐mediated pathway increases the expression level of the transcription factors ATF3 and ATF4 ^27–29^, subsequently forming the ATF3/ATF4 complex to induce the transcription of DDIT3 (CHOP), which finally results in apoptosis^30,31^. Although EGFR-TKIs have been reported to induce ER stress response^32^, the association between EGFR-TKI resistance and ER stress in cancer cells has not yet been elucidated.

EphA3 belongs to the Eph receptor-tyrosine kinases (RTKs), regulates cell–cell interaction, and has a role in development and tissue organization^33^. Downstream signaling of EphA3 is induced by its preferential binding partner, ephrin-A5, followed by activation of GTPase or ERK and dephosphorylation of AKT, resulting in cytoskeletal re-organization, cell retraction, and differentiation^34^. EphA3 was first identified as a cell surface antigen on a pre-B lymphoblastic leukemia cell line^35^, and was then found to be an antigen on melanoma cells recognized by lytic CD4^+^ T cells^36^. EphA3 is overexpressed in various tumor cells and is implicated in the maintenance of tumor-initiating cells in glioblastoma and leukemia^33,34^. Frequent somatic mutations of EphA3 as an RTK have been shown in various metastatic cancers, suggesting its role as a tumor suppressor^37^. In breast cancer, expression levels of EphA3 vary depending on the disease stage, being highly expressed in lymph node metastases^38^. However, very little is known regarding the potential role of EphA3 in the pathophysiology of breast cancer, including TNBC.

In the present study, we focused on the role of IL-26 in regulating the effect of EGFR-TKI in TNBC. We found that human TNBC is exposed to IL-26 in the TME, and that IL-26 activates the bypass pathway of EGFR-TKI, while blockade of IL-26 overcomes EGFR-TKI resistance in TNBC. Moreover, we determined that the interaction of IL-26 with EphA3 on TNBC cells inhibits the signaling pathway of ER stress via cross-talk with EGFR signaling. Our findings demonstrate the critical role of IL-26 in mediating EGFR-TKI resistance in TNBC, and suggest a potential novel therapeutic strategy for TNBC involving the combination of anti-IL26 and anti-EGFR agents.

## Results

### Expression of IL-26 in tumor-infiltrating lymphocytes (TILs) in clinical specimens of TNBC

**Fig. 1:**
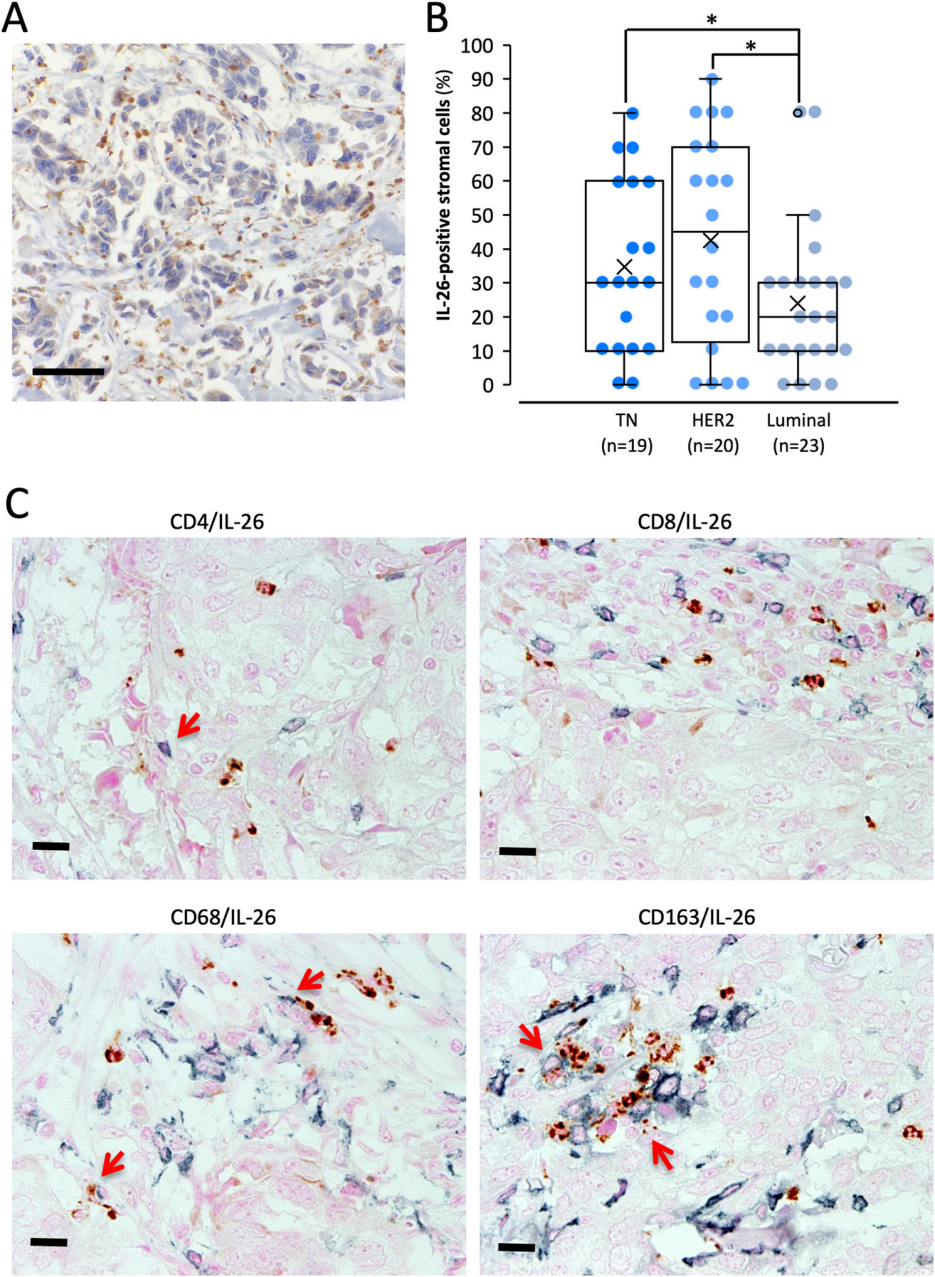
Immunohistochemistry of breast carcinoma clinical specimens. **A** TNBC tissue specimens were stained with anti-human IL-26 mAb (*n* = 19). All immunohistochemistry specimens were counterstained with hematoxylin. A representative image of the TNBC case with high IL-26 protein expression was shown. Original magnification, ×100. Scale bar, 50 μm. **B** Each subtype of breast carcinoma tissue specimen was stained with anti-human IL-26 mAb (TNBC (*n* = 19), HER2 (*n* = 20), and Luminal (*n* = 23)). IL-26 in cytoplasm staining of stromal immune cells was determined semi-quantitatively in 10% increments. The percentage of IL-26-positive stromal immune cells in each subtype of breast carcinoma is shown. *p < 0.05. **C** TNBC tissue specimens (the same case as shown in **A**) were double-stained with IL-26 (brown) plus CD4, CD8, CD68, or CD163 (blue), respectively. All tissue sections were counterstained with hematoxylin. IL-26 was merged with cell surface CD4, CD68, and CD163 (arrows in each panel). Original magnification, ×40. Scale bar, 25 μm.

Our evaluation of IL-26 expression in clinical specimens by immunohistochemistry showed that IL-26 was clearly detected in TILs of TNBC (Fig. 1A), as well as HER2 and Luminal tumors (Supplementary Fig. S1). Although IL-26-positive TILs were observed in all subtypes, the mean percentage of IL-26-positive TILs in TNBC and HER2 type was significantly higher than luminal tumors (Fig. 1B). Immunohistochemical analysis revealed that CD4^+^ T cells, CD68^+^ M1 macrophages, and CD163^+^ M2 macrophages in the TME expressed IL-26 (Fig. 1C). These data suggest that TNBC cells are exposed to IL-26 secreted by CD4^+^ T cells and macrophages infiltrating in the TME.

### Exogenous IL-26 activates bypass pathway of EGFR-TKI in mouse and human TNBC

**Fig. 2:**
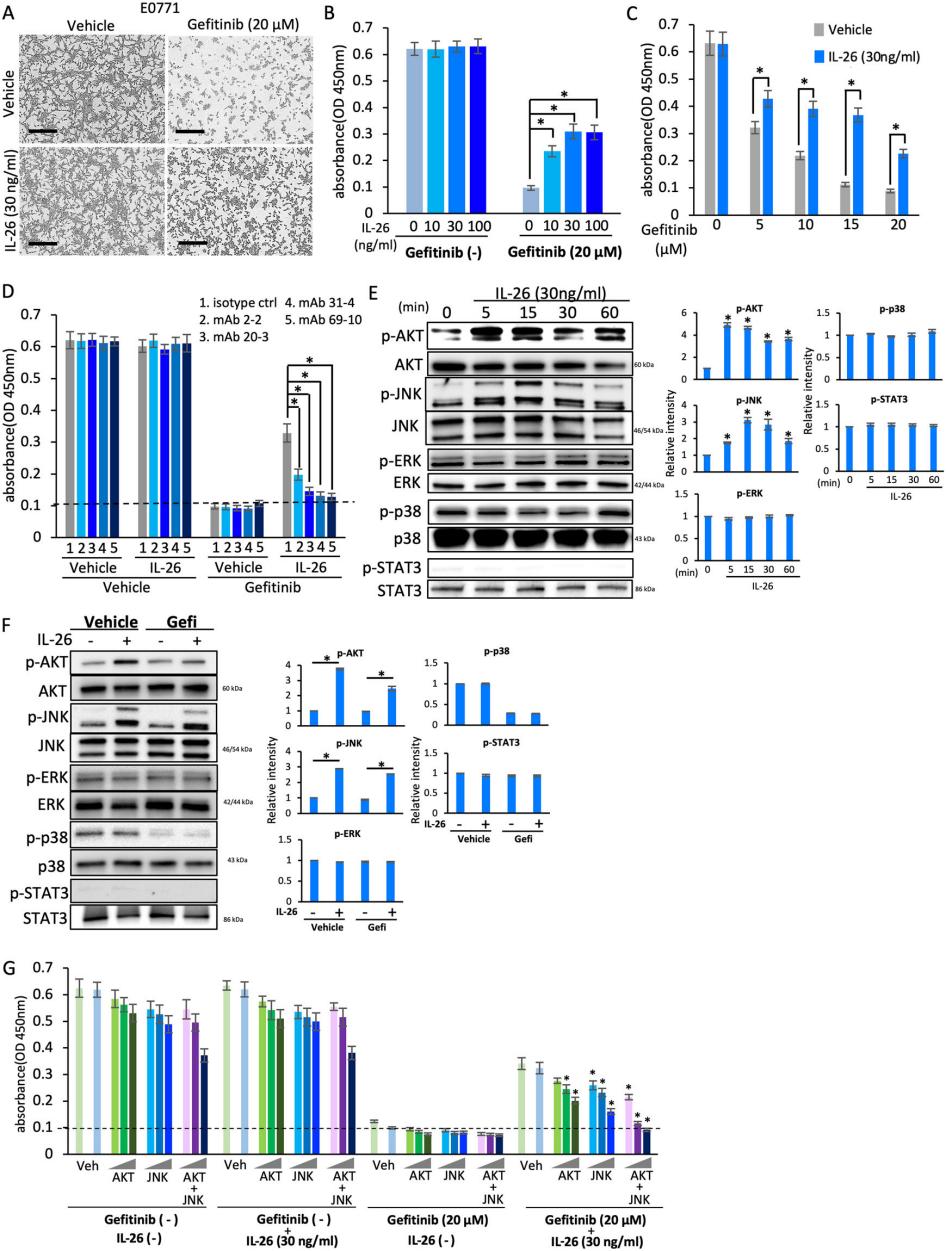
Exogenous IL-26 activates bypass pathway of EGFR-TKI in mouse TNBC in in vitro assays. **A** Phase contrast microscopy of E0771 cells (1 × 10^4^) following 48 h incubation with IL-26 or control vehicle in the presence or absence of gefitinib. Original magnification, ×100. Scale bar, 100 μm. Data shown are representative images of five independent experiments with similar results. **B** E0771 cells were treated with the indicated dose of IL-26 in the presence or absence of gefitinib (20 μM) for 48 h. *p < 0.01. **C** E0771 cells were treated with IL-26 (30 ng/ml) in the presence of various doses of gefitinib (5, 10, 15, or 20 μM). *p < 0.01. **D** E0771 cells were treated with IL-26 (30 ng/ml) and/or gefitinib (20 μM) in the presence of anti-IL-26 neutralizing mAb or isotype control mouse IgG (20 µg/ml, each) for 48 h. The dashed line is the standard value of gefitinib plus vehicle. **E** E0771 cells were stimulated with IL-26 (30 ng/ml) for the indicated periods, and then submitted to Western blot analysis using anti-phosphorylated AKT, JNK, ERK, p38, and STAT3 antibodies, and reblotting with anti-pan AKT, JNK, ERK, p38, and STAT3 antibodies. **F** E0771 cells were stimulated with IL-26 (30 ng/ml) in the presence or absence of gefitinib (20 μM) for 15 min, and then submitted to Western blot analysis as described in **E**. **G** E0771 cells were treated with IL-26 (30 ng/ml) and/or gefitinib (20 μM) in the presence or absence of various concentrations of signal inhibitors (AKT inhibitor, JNK inhibitor or combination of AKT inhibitor and JNK inhibitor) for 48 h. The dashed line is the standard value of gefitinib plus vehicle. *p < 0.01. **B**–**D**, **G** Cell proliferation was assessed by MTT assay. Representative data of five (**B**) and three (**C**, **D**, **G**) independent experiments are shown as mean ± S.D. of triplicate samples, and similar results were obtained in each experiment. **E**, **F** Data shown are representative of five independent experiments, and similar results were obtained in each experiment. Band intensity of phospho-proteins was normalized to the appropriate pan proteins, and relative intensity compared with unstimulated cells is shown as mean ± SEM from five independent experiments. *p < 0.01.

We next evaluated the effects of exogenous IL-26 in combination with EGFR-TKI treatment on murine TNBC cell line E0771 proliferation. Gefitinib treatment suppressed E0771 cell proliferation, while exogenous IL-26 significantly inhibited gefitinib-induced suppression of cell proliferation in a dose-dependent manner of IL-26 (Fig. 2A, B). In addition, exogenous IL-26 significantly inhibited gefitinib-induced suppression of cell proliferation at higher doses of gefitinib (Fig. 2C). Meanwhile, treatment with IL-26 alone had no effect on cell proliferation of E0771 (Fig. 2A–C). Moreover, four different house-made anti-IL-26 neutralizing mAbs, clones 2-2, 20-3, 31-4, and 69-10, reversed the effect of IL-26 on gefitinib-induced suppression of cell proliferation (Fig. 2D). Investigating specific signaling pathway mediators, we found that exogenous IL-26 activated AKT and JNK phosphorylation but not STAT3 in E0771 cells (Fig. 2E). Moreover, the addition of exogenous IL-26 resulted in activation of AKT and JNK despite treatment with gefitinib (Fig. 2F). Furthermore, inhibition of both AKT and JNK resulted in the marked diminution of IL-26-stimulated proliferation, while inhibition of either AKT or JNK led to partially reduced cell proliferation (Fig. 2G). Similar results were demonstrated with human TNBC cells as obtained with the murine TNBC cells (Supplementary Figs. S2, S3). In addition, exogenous IL-26 significantly inhibited the suppressive effect of erlotinib, another EGFR-TKI, on proliferation of the murine and human TNBC cell lines E0771, HCC70, and MDA-MB468, at similar levels to those seen with gefitinib (Supplementary Fig. S4). These findings indicate that IL-26 induces increased phosphorylation of AKT and JNK, hence activating the EGFR-TKI-associated bypass pathway to result subsequently in tumor growth of both murine and human TNBC.

### Interaction of IL-26 and EphA3 in murine TNBC cells

**Fig. 3:**
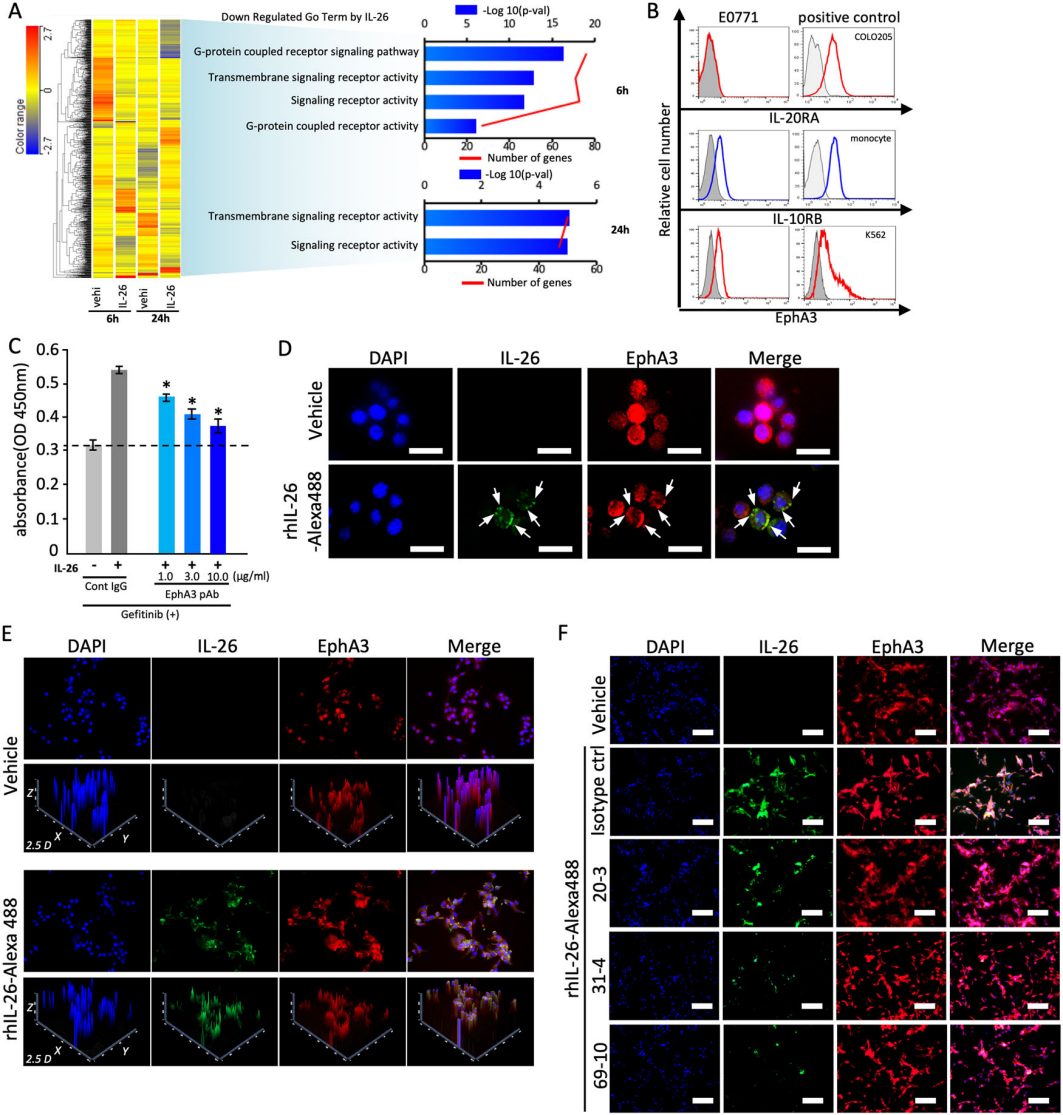
IL-26 activates bypass pathway of EGFR-TKI via its EphA3 receptor in mouse TNBC cells. **A** E0771 cells were treated with IL-26 (30 ng/ml) or control vehicle (vehi) for 6 or 24 h, and total RNA was isolated and subjected to DNA microarray analysis as described in the Materials and Methods. Left: A heat map of 960 genes differentially (fold change ≥ 2.0) expressed between vehicle- and IL-26-treated E0771 cells was constructed by hierarchical cluster analysis. Right: significantly downregulated GO terms in IL-26-induced genes. **B** E0771 cells and the indicated positive control cells were stained with each Ab. Data are shown as histogram of IL-20RA, IL-10RB, and EphA3, and the gray area in each histogram shows isotype control data. **C** E0771 cells were treated with IL-26 (30 ng/ml) and gefitinib (20 μM) in the presence of the indicated doses of anti-EphA3 pAb or control IgG for 48 h. Cell proliferation was assessed by MTT assay. The dashed line is the standard value of gefitinib plus vehicle. Data are shown as mean ± S.D. of triplicate samples. *p < 0.01. **D** E0771 cells were treated with Alexa Fluor 488-labeled recombinant human IL-26 (rhIL-26-Alexa488 (green)) (30 ng/ml) for 1 h, followed by immunostaining with anti-mouse EphA3 pAb (red) and DAPI (blue). IL-26 was merged with cell surface EphA3 (arrows in lower panel). Original magnification, ×200. Scale bar, 20 μm. **E** Reconstructed 2.5D image shows the intensity of fluorescent peaks of the images from **D**. 2.5D intensity plot of IL-26, EphA3, and DAPI signals represents absolute signal intensities of each pixel. Merged views and individual channels were shown. The 2.5D intensity plots show that IL-26 and EphA3 almost equally distributed and frequently colocalized at the rim. Original magnification, ×200. **F**. E0771 cells were treated with rhIL-26-Alexa488 (green) (30 ng/ml) in the presence of anti-IL-26 mAb (clone 20-3, 31-4 or 69-10) or control IgG (50 µg/ml, each) for 1 h, followed by immunostaining with anti-mouse EphA3 pAb (red) and DAPI (blue). Original magnification ×200. Scale bar, 50 μm. **B**–**F** Data shown are representative of three independent experiments with similar results.

IL-26 stimulation did not induce STAT3 phosphorylation in E0771 cells, suggesting the existence of a novel receptor/signaling pathway other than IL-20RA/IL-10RB-STAT3. To identify the novel interacting proteins of IL-26 in TNBC cells, we performed pathway analysis by DNA microarray of E0771 cells treated with IL-26 (Fig. 3A). The genes related to transmembrane signaling receptor activity and downstream signaling receptor activity were downregulated in E0771 cells treated with IL-26 for both 6 and 24 h. Among them, IL-26 significantly reduced the expression level of EphA3 and altered the downstream signaling of EphA3, indicating the engagement of the EphA3 receptor signaling pathway in IL-26-treated E0771 cells (GEO accession: GSE147804). Of note is the fact that the expression of EphA3 was detected in E0771 (Fig. 3B). On the other hand, while IL-10RB expression was detected, expression of IL-20RA, a major IL-26 binding chain, was not observed in E0771 (Fig. 3B). Blockade of EphA3 by anti-EphA3 pAb resulted in significant inhibition of IL-26-mediated cell proliferation in the setting of EGFR-TKI treatment, in an anti-EphA3 pAb dose-dependent manner (Fig. 3C). Immunocytochemical analysis revealed that exogenous IL-26 was colocalized with cell surface EphA3 (Fig. 3D, E), and that anti-IL-26 mAbs inhibited EphA3/IL-26 interaction (Fig. 3F). Moreover, this colocalization was inhibited by the addition of soluble EphA3-Ig (Supplementary Fig. S5). Taken together, these data suggest that IL-26 interacts with EphA3, but not with the known receptor IL-20RA, on TNBC cells.

### Interaction of IL-26 and EphA3 in human TNBC cells

**Fig. 4:**
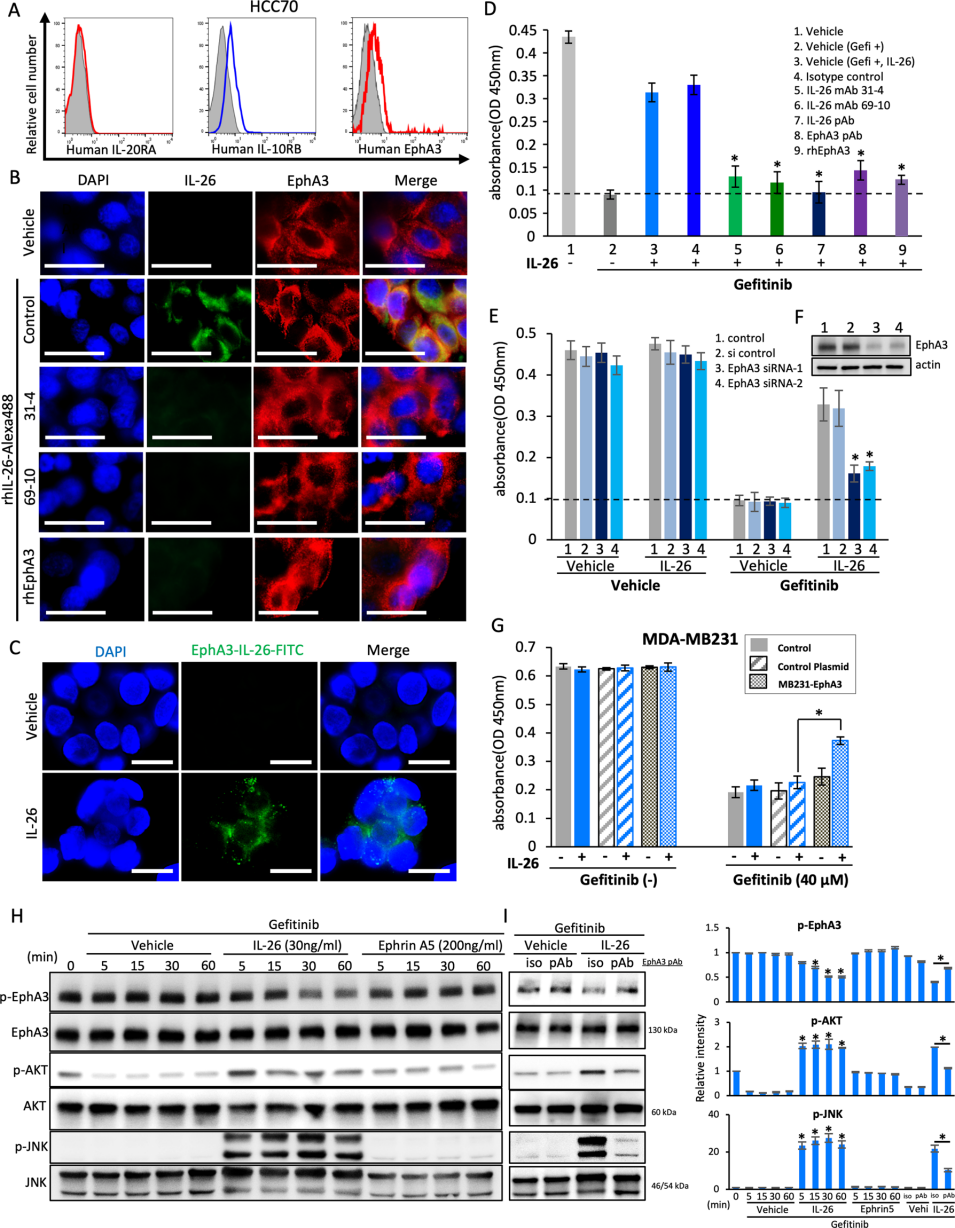
IL-26 activates bypass pathway of EGFR-TKI via its EphA3 receptor in human TNBC cells. **A** HCC70 cells were stained with each Ab. Data are shown as histogram of IL-20RA, IL-10RB, and EphA3, and the gray area in each histogram shows isotype control data. **B** HCC70 cells were treated with Alexa Fluor 488-labeled recombinant human IL-26 (rhIL-26-Alexa488 (green)) (30 ng/ml) in the presence of anti-IL-26 mAb (clone 31-4 or 69-10), recombinant human EphA3-Ig (rhEphA3) or control IgG (50 µg/ml, each) for 1 h, followed by immunostaining with anti-human EphA3 pAb (red) and DAPI (blue). Original magnification, ×200. Scale bar, 20 μm. **C** HCC70 cells were treated with recombinant human IL-26 (30 ng/ml) for 1 h, followed by immunostaining and in situ PLA detection of IL-26-EphA3 interaction (green). Cell nuclei are counterstained with DAPI (blue). Original magnification, ×200. Scale bar, 20 μm. **D** HCC70 cells were treated with IL-26 (30 ng/ml) and gefitinib (40 µM) in the presence of the indicated Abs or rhEphA3-Ig (50 µg/ml, each) for 48 h. Data are shown as mean ± S.D. of triplicate samples. *p < 0.01. **E**, **F** HCC70 cells were transfected with siRNA and stimulated with IL-26 (30 ng/ml) in the presence or absence of gefitinib (40 µM) for 48 h. The expression level of EphA3 was confirmed by western blotting (**F**). Data are shown as mean ± S.D. of triplicate samples. *p < 0.01. **G** MDA-MB231 cells (EphA3-negative) were transfected with EphA3 plasmid and stimulated with IL-26 (30 ng/ml) in the presence or absence of gefitinib (40 µM) for 48 h. The expression level of cell surface EphA3 was confirmed by Flow cytometry (Supplementary Fig. S8). Data are shown as mean ± S.D. of triplicate samples. *p < 0.01. **H** HCC70 cells were treated with IL-26 (30 ng/ml) or Ephrin A5 (200 ng/ml) in the presence of gefitinib (40 µM) for the indicated periods, and then submitted to western blot analysis using anti-phosphorylated EphA3, AKT, and JNK antibodies, and reblotting with anti-pan EphA3, AKT, and JNK antibodies. **I**. HCC70 cells were treated with IL-26 (30 ng/ml) and gefitinib (40 µM) in the presence of anti-EphA3 pAb or isotype control IgG (50 µg/ml, each) for 15 min, and then submitted to Western blot analysis as described in **H**. **A**–**I** Data shown are representative of three independent experiments with similar results. **D**, **E**, **G** Cell proliferation was assessed by MTT assay. The dashed line is the standard value of gefitinib plus vehicle. **H**, **I** Band intensity of phospho-proteins was normalized to the appropriate pan proteins, and relative intensity compared with unstimulated cells is shown as mean ± SEM from three independent experiments. *p < 0.01.

Expression of EphA3 was also detected in both human TNBC cell lines and TNBC cells of human clinical tissue samples (Fig. 4A and Supplementary Figs. S6, S7). On the other hand, while IL-10RB expression was detected, expression of IL-20RA was not observed (Fig. 4A and Supplementary Fig. S6). Immunocytochemical analysis revealed that exogenous IL-26 was colocalized with cell surface EphA3 (Fig. 4B). Meanwhile, anti-IL-26 mAbs inhibited EphA3/IL-26 interaction, and this colocalization was also inhibited by the addition of soluble EphA3-Ig (Fig. 4B). We next performed pull-down assays utilizing recombinant tagged IL-26 and EphA3 proteins, and immunoprecipitation assays with cell lysates. However, direct protein-protein interaction involving IL-26 and EphA3 could not be clearly observed due to the non-specific binding of IL-26 to tag proteins (data not shown). Meanwhile, in situ proximity‐ligation assay (PLA) revealed IL-26 and EphA3 existed in close proximity (Fig. 4C). Moreover, blockade of EphA3 by anti-EphA3 pAb or soluble EphA3-Ig resulted in significant inhibition of IL-26-mediated cell proliferation in the setting of EGFR-TKI treatment, at similar levels to those obtained with anti-IL-26 antibody blockade (Fig. 4D). Furthermore, genetical ablation of EphA3 expression by siRNA resulted in significant inhibition of IL-26-mediated cell proliferation in the setting of EGFR-TKI treatment (Fig. 4E, F). In addition, IL-26-mediated EGFR-TKI resistance was not clearly observed in human TNBC cell line MDA-MB231, which hardly expressed EphA3 (Supplementary Fig. S8), whereas genetical EphA3 overexpression by transfection of EphA3-containing plasmid resulted in the acquisition of IL-26-mediated EGFR-TKI resistance (Fig. 4G). The addition of exogenous IL-26 to HCC70 cells in the presence of gefitinib resulted in dephosphorylation of EphA3 and activation of AKT and JNK phosphorylation, results not observed following stimulation with Ephrin A5, a known ligand for EphA3 (Fig. 4H). On the other hand, dephosphorylation of EphA3 and activation of AKT and JNK phosphorylation were partially inhibited by treatment with anti-EphA3 pAb (Fig. 4I). Taken together, these findings indicate that interaction of IL-26 and EphA3 induces increased phosphorylation of AKT and JNK not only in murine TNBC but also in human TNBC, hence activating the EGFR-TKI-associated bypass pathway to result subsequently in tumor growth.

### IL-26 interaction with EphA3 suppresses EGFR-TKI-induced ER stress signaling pathway in TNBC cells

**Fig. 5:**
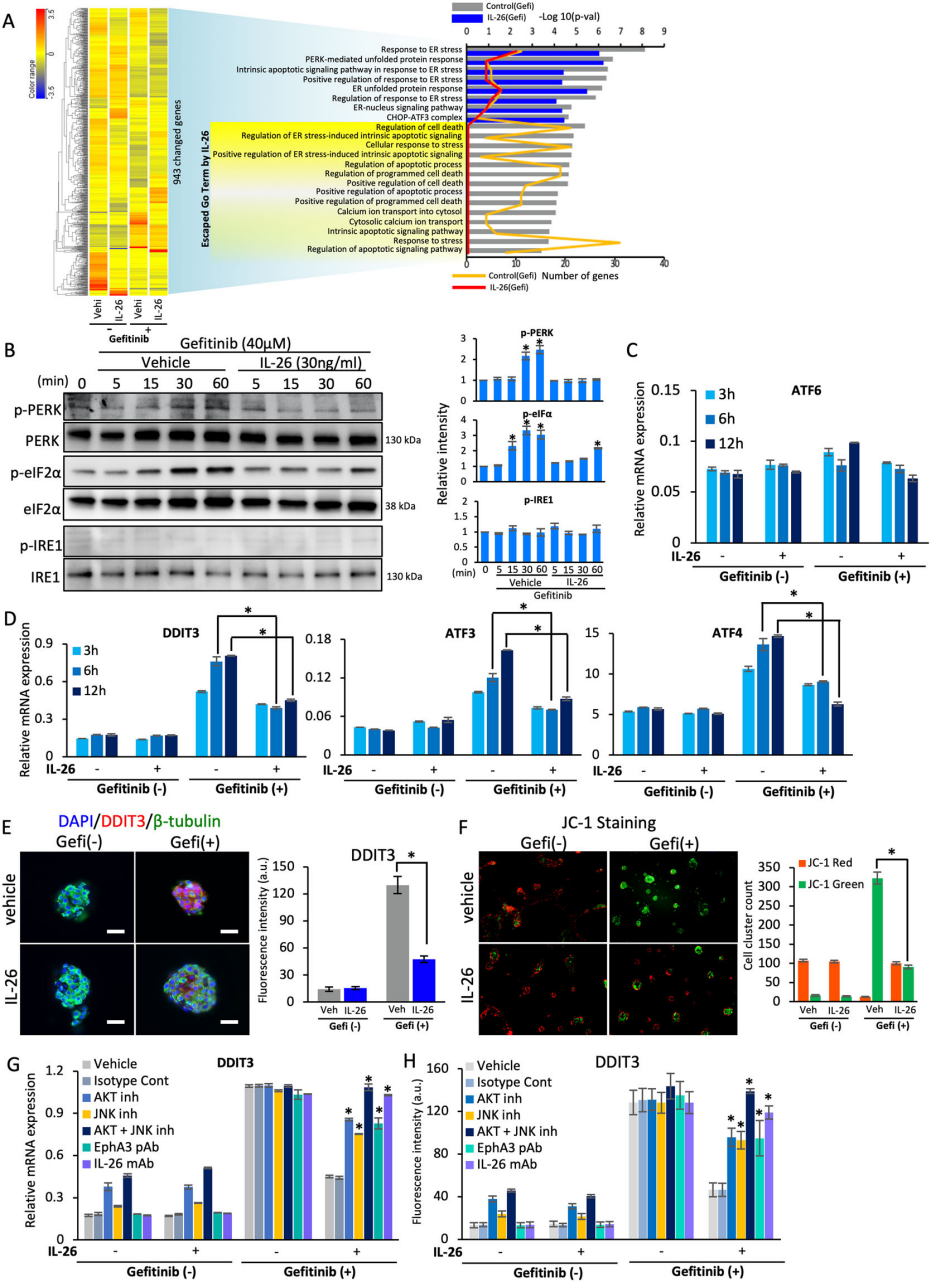
IL-26 suppresses EGFR-TKI-induced ER stress in TNBC cells. **A** HCC70 cells were treated with IL-26 (30 ng/ml) or control vehicle (vehi) in the presence or absence of gefitinib (40 μM) for 3 h. Total RNA was isolated and subjected to DNA microarray analysis as described in the “Materials and methods” section. Left: A heat map of 943 genes differentially (fold change ≥ 2.0) expressed between gefitinib plus IL-26- and gefitinib alone-treated HCC70 cells was constructed by hierarchical cluster analysis. Right: significantly enriched Gene Ontology (GO) terms in gefitinib-induced and IL-26-reduced genes. **B** HCC70 cells were treated with IL-26 (30 ng/ml) in the presence of gefitinib (40 µM) for the indicated periods, and then submitted to Western blot analysis using anti-phosphorylated PERK, eIF2α, and IRE1 antibodies, and reblotting with anti-pan PERK, eIF2α, and IRE1 antibodies. Data shown are representative of three independent experiments, and similar results were obtained in each experiment. Band intensity of phospho-proteins was normalized to the appropriate pan proteins, and relative intensity compared with unstimulated cells is shown as mean ± SEM from three independent experiments. * *p* < 0.01. **C**, **D** HCC70 cells were treated with IL-26 (30 ng/ml) in the presence or absence of gefitinib (40 μM) for the indicated periods. mRNA expression levels of ATF6 (**C**) or DDIT3, ATF3, and ATF4 (**D**) were quantified by qRT-PCR. *p < 0.01. **E** HCC70 cells were treated with IL-26 (30 ng/ml) in the presence or absence of gefitinib (40 μM) for 24 h, followed by immunostaining with anti-human DDIT3 pAb (red), β-tubulin (green), and DAPI (blue). Original magnification, ×400. Scale bar, 20 μm. **F** HCC70 cells were treated with IL-26 (30 ng/ml) in the presence or absence of gefitinib (40 μM) for 24 h, followed by JC-1 staining. Red fluorescence, sign of preserved mitochondrial membrane potential (ΔΨ_m_), was observed in vehicle- or IL-26-treated HCC70 cells in the absence of gefitinib, whereas green fluorescent signals, index of mitochondrial membrane depolarization were prominently observed in vehicle-treated HCC70 cells in the presence of gefitinib. Stained cell clusters were quantitated using Image-J software. *p < 0.01. **G** HCC70 cells were treated with IL-26 (30 ng/ml) and/or gefitinib (40 μM) in the presence of vehicle, signal inhibitors, anti-EphA3 pAb or anti-IL-26 mAb (clone 69-10) (50 µg/ml, each) for 6 h. mRNA expression levels of DDIT3 were quantified by qRT-PCR. *p < 0.01. **H** HCC70 cells were treated as described in G for 24 h. Expression of DDIT3 was detected by Immunofluorescence staining with anti-human DDIT3 pAb. Fluorescence intensity and stained cell clusters were quantitated using Image-J software. *p < 0.01. **C**–**H** Representative data of three independent experiments are shown as mean ± S.D. of triplicate samples, and similar results were obtained in each experiment.

To identify a key regulator of tumor growth downstream of IL-26/EphA3 interaction in TNBC, we performed pathway analysis by DNA microarray of HCC70 cells treated with IL-26 and gefitinib (Fig. 5A). This work revealed a significant reduction in the expression levels of ER stress-associated molecules, indicating the engagement of the ER stress signaling pathway in HCC70 cells treated with IL-26 and gefitinib (GEO accession: GSE171641). To confirm these in silico findings, we examined the role of IL-26/EphA3 in the EGFR-TKI-related ER stress pathway. For this purpose, we analyzed the activation of three major ER stress signal transducers, PERK, IRE1, and ATF6, in TNBC cells treated with IL-26 and gefitinib. Although the PERK-eIF2α pathway was enhanced following gefitinib treatment, phosphorylation of IRE1 and upregulation of ATF6 expression were hardly observed in HCC70 cells stimulated with gefitinib (Fig. 5B, C). Of note, stimulation with gefitinib plus IL-26 significantly decreased gefitinib-induced phosphorylation of PERK and eIF2α in HCC70 (Fig. 5B). Moreover, we analyzed expression levels of markers for ER stress signaling, DDIT3, ATF3, and ATF4, in TNBC cells treated with IL-26 and gefitinib. mRNA expression levels of DDIT3, ATF3, and ATF4 were clearly decreased by IL-26 treatment with gefitinib (Fig. 5D). Similarly, in other TNBC cell lines, MDA-MB468 and E0771, mRNA expression levels of DDIT3, ATF3, and ATF4 were significantly decreased by IL-26 treatment with gefitinib (Supplementary Fig. S9). Furthermore, other ER stress-associated genes including IL-6, IL-8, and CXCL2 were significantly decreased in HCC70, MDA-MB468, and E0771 by IL-26 treatment with gefitinib (Supplementary Fig. S10). Immunocytochemistry also revealed a significant decrease in the expression level of DDIT3 (Fig. 5E). In addition, a reduction in mitochondrial activity was significantly inhibited by IL-26 and gefitinib (Fig. 5F). While a decrease in the expression level of DDIT3 mRNA was significantly inhibited by treatment with AKT inhibitor alone, JNK inhibitor alone, or EphA3 pAb, this inhibition was more pronounced following treatment with both AKT and JNK inhibitors, comparable to anti-IL-26 mAb treatment (Fig. 5G). Similarly, immunocytochemistry revealed a significant decrease in the expression level of DDIT3 by treatment with AKT inhibitor, a JNK inhibitor, EphA3 pAb, and anti-IL-26 mAb (Fig. 5H). Moreover, similar results were also observed in mitochondrial activity (Supplementary Fig. S11). Taken together, these results indicate that phosphorylation of AKT and JNK has an important role in ER stress response, particularly the PERK-eIF2α-DDIT3 pathway, to induce cell survival and proliferation in TNBC treated with IL-26 in the presence of gefitinib.

### IL-26 enhances in vivo TNBC tumor growth in EGFR-TKI-treated murine models

**Fig. 6:**
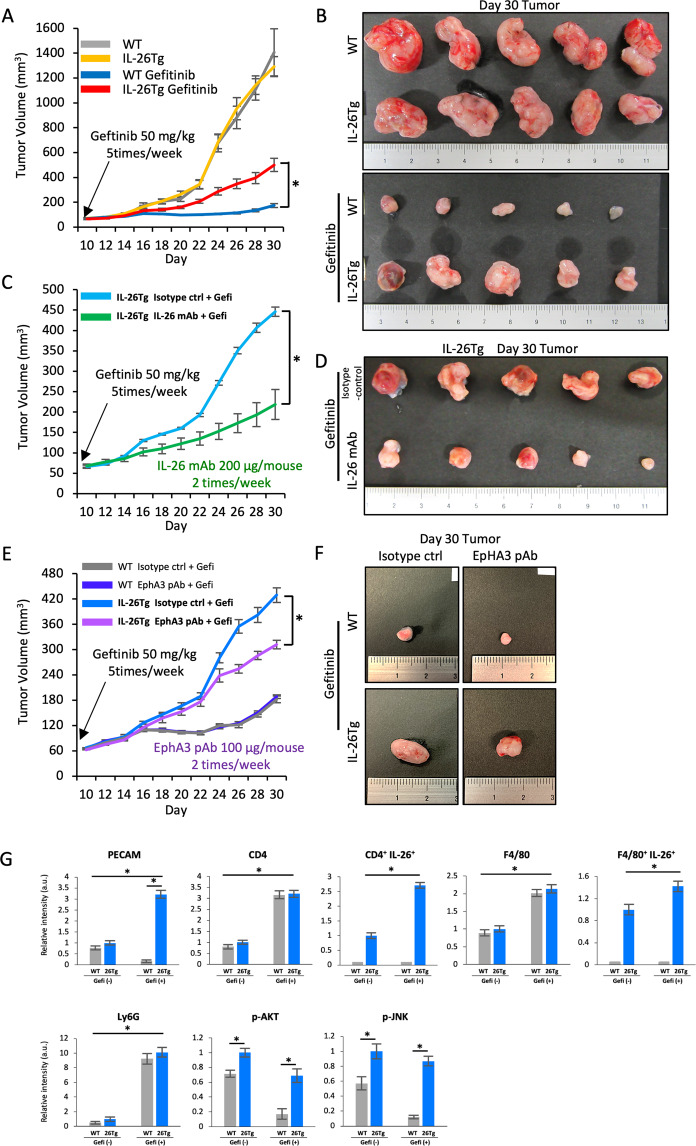
IL-26 promotes in vivo tumor growth against EGFR-TKI. **A** E0771 cells (5 × 10^5^) with Matrigel were injected subcutaneously into the flank of hIL-26Tg (IL-26Tg) or littermate control mice (WT) (each, *n* = 8). Gefitinib was administered by oral gavage (50 mg/kg) once a day five times a week for the duration of the study from 10 days after E0771 cell inoculation. *n* = 8 mice for each group at each time point. mean ± SEM of each group. *p < 0.01. **B** Macroscopic manifestation of the tumors in experiment A. Tumors were resected at 30 days after inoculation. **C** E0771 cells (5 × 10^5^) with Matrigel were inoculated subcutaneously into the flank of hIL-26Tg mice, and gefitinib was administered by the same method as described in experiment A. IL-26 mAb (clone 69-10) or mouse IgG isotype control (each, 200 µg/dose) was subsequently injected intraperitoneally once a day twice a week from 10 days after E0771 inoculation. *n* = 8 mice for each group at each time point. mean ± SEM of each group. *p < 0.01. **D** Macroscopic manifestation of the tumors in the experiment C. Tumors were resected at 30 days after inoculation. **E** E0771 cells (5 × 10^5^) with Matrigel were inoculated subcutaneously into the flank of hIL-26Tg or control mice, and gefitinib was administered by the same method as described in experiment A. Anti-mouse EphA3 pAb or goat IgG isotype control (each, 100 µg/dose) was subsequently injected intraperitoneally once a day twice a week from 10 days after E0771 inoculation. *n* = 8 mice for each group at each time point. mean ± SEM of each group. *p < 0.01. **F** Macroscopic manifestation of the tumors in the experiment E. Tumors were resected at 30 days after inoculation. **G** Immunofluorescence staining of tumor specimens resected at 17 days after E0771 inoculation. Representative images are shown in Supplementary Fig. S13. Data are shown as mean ± S.E. of relative fluorescence intensity from 8 mice for each group, comparing values in vehicle-treated WT mice, gefitinib-treated WT mice, or hIL-26Tg mice to those in vehicle-treated WT mice (*p < 0.01).

To extend the in vitro findings above to in vivo experimental systems, we next conducted in vivo tumor growth assay of TNBC following gefitinib treatment, utilizing hIL-26Tg mice. E0771 tumors were grown in hIL-26Tg and its littermate control mice (yellow and gray lines in Fig. 6A and tumor photos of upper panel in Fig. 6B), and tumor growth was suppressed in control mice following treatment with gefitinib (blue line in Fig. 6A and upper tumor photos of the lower panel in Fig. 6B). Meanwhile, tumor suppression by gefitinib was significantly inhibited in hIL-26Tg mice (red line in Fig. 6A and lower tumor photos of the lower panel in Fig. 6B). Moreover, tumor growth was significantly decreased in TNBC transplanted in hIL-26Tg mice following administration of anti-IL-26 mAb and gefitinib (green line in Fig. 6C), compared to that seen with control mAb and gefitinib (pale blue line in Fig. 6C). Of note is that treatment with anti-IL-26 mAb alone had no effect on tumor growth (green line in Supplementary Fig. S12A). Furthermore, tumor growth was significantly decreased in TNBC transplanted in hIL-26Tg mice following administration of anti-EphA3 pAb and gefitinib (purple line in Fig. 6E), compared to that seen with control Ab and gefitinib (pale blue line in Fig. 6E). Of note is that treatment with anti-EphA3 pAb alone had no effect on tumor growth (green line in Supplementary Fig. S12B).

Immunohistochemical assay revealed that CD4^+^ T cells and F4/80+ macrophages in the TME expressed IL-26 (Fig. 6G and Supplementary Fig. S13C, D). Moreover, granulocyte levels in the TME of both hIL-26Tg and control mice treated with gefitinib were increased (Fig. 6G and Supplementary Fig. S13E), possibly with response to KC and MIP-2 induced by ER stress signaling (shown in Supplementary Fig. S10). PECAM-positive blood vessels were markedly increased in gefitinib-treated hIL-26Tg mice (Fig. 6G and Supplementary Fig. S13B). Finally, cells with phosphorylated AKT and JNK were clearly increased in gefitinib-treated hIL-26Tg mice as compared with gefitinib-treated control mice (Fig. 6G, Supplementary Fig. S13F, G). Taken together, our data suggest that TNBC cells are exposed to IL-26 secreted by CD4^+^ T cells and macrophages infiltrating in the TME, evoking a resistance to EGFR-TKI therapy in TNBC, associated with phosphorylation of AKT and JNK of ER stress response.

**Fig. 7:**
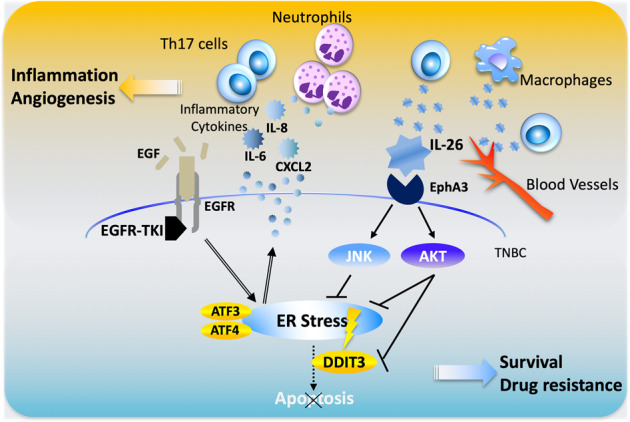
Hypothetical schema of the effect of IL-26 on EGFR-TKI resistance. IL-26 activates AKT and JNK signaling of bypass pathway of EGFR-TKI via interaction with its newly identified IL-26 functional receptor EphA3, leading to suppression of EGFR-TKI-induced ER stress, which subsequently results in TNBC tumor survival and drug resistance. Moreover, production of ER stress-associated inflammatory cytokines/chemokines including IL-6, IL-8, and CXCL2 was enhanced following treatment with EGFR-TKI, leading to the recruitment of neutrophils in TME, which further promotes the inflammation and recruitment of other inflammatory cells such as T cells and macrophages.

Based on our experimental findings, Fig. 7 depicts a schematic of the inhibitory effect of IL-26 on EGFR-TKI-mediated tumor suppression in TNBC cells.

## Discussion

Originally identified in *Herpes saimiri*-infected T cells as AK155 ^39^, IL-26 has an important role as a mediator of local inflammation^11,12^. Our group has revealed novel functions of IL-26 in various in vivo models utilizing humanized mice or hIL-26Tg mice, and succeeded in developing anti-IL-26 neutralizing mAbs to establish a novel IL-26-targeted therapy^13,40–42^. We recently demonstrated that IL-26 functions as an angiogenic factor equivalent to VEGF, strongly suggesting a role of IL-26 in angiogenesis in the TME and malignant progression. While IL-26 is involved in cancer biology^16,17,43,44^, the precise molecular mechanisms associated with this process, including the identification of the functional receptor of IL-26 in cancer cells and its downstream signaling events, are not yet clarified.

Our present study demonstrates that IL-26 has a critical role in cell growth of EGFR-TKI-treated TNBC via activation of an EGFR bypass pathway. By exploring the mechanisms involved in IL-26-mediated EGFR-TKI resistance in TNBC, we found that the interaction of IL-26 and EphA3 suppresses ER stress signaling pathway induced by EGFR-TKI treatment. Previous work showed that EGFR-TKI‐treatment is linked to the induction of ER stress response^32^, and erlotinib‐induced ER stress signaling can promote survival of EGFR-TKI‐persister cells through transcriptional adaptation^45^. While intense ER stress induces cell death, feeble ER stress promotes the survival of tumor cells. Previous work showed that ER stress-mediated activation of the IRE1α pathway is a key factor for tumor-initiating cell survival in breast cancer^45,46^. However, the effects of intense ER stress through the PERK-ATF4-DDIT3 pathway on breast cancer, and the mechanism by which breast cancer cells subjected to intense ER stress escape ER stress-induced cell death, have remained incompletely characterized. Our study revealed that interaction of IL-26 and EphA3 in TNBC activates AKT and JNK, leading to tumor proliferation by inhibiting EGFR-TKI-provoked PERK-eIF2α-DDIT3 pathway and ER stress-associated cell death. While EGFR is highly expressed on most TNBC, activating mutations are rarely observed in the EGFR expressed on TNBC^9^. Our present data strongly suggest that EGF-wild-type EGFR signaling has an important role in the survival or growth of TNBC, and that EGFR-TKI acting through this wild-type EGFR induces intense ER stress. Future studies are needed to define the precise role of EGFR in TNBC and the effect of EGFR-TKI on TNBC, in comparison to the role of well-known EGFRs with activating mutations expressed on other neoplasms such as lung or colon cancers. ER stress in cancer cells is associated with the production of various proinflammatory molecules^21,47^. Our data showed that gefitinib-treated TNBC cells exhibited enhanced production of proinflammatory cytokines/chemokines such as IL-6, IL-8, and CXCL2, hence increasing the number of tumor-infiltrating immune cells including IL-26-producing cells in the TME. Recent evidence showed that IL-26 promotes an inflammatory TME of TNBC, at least partially through binding to neutrophil extracellular traps (NET) DNA to stimulate the expression of multiple proinflammatory cytokines in the TME, which collectively contribute to engraftment, tumor growth, and metastasis of TNBC^44^. Since granulocyte levels in the TME of hIL-26Tg mice treated with gefitinib were increased (Fig. 6G and Supplementary Fig. S13E), further studies are needed to clarify whether IL-26 affects the production of inflammatory cytokines from immune cells infiltrating the TME of TNBC treated with gefitinib, and whether these effects of IL-26 influence tumor growth or metastasis of TNBC in our in vivo model.

The previous report showed that IL-26 binds to a cell surface heterodimeric receptor IL-20RA/IL-10RB, inducing downstream signaling cascade events via STAT3 phosphorylation^19^. However, the TNBC cell lines used in the present study did not express IL-20RA. These findings strongly suggest that the downstream signaling events in TNBC following IL-26 stimulation were not mediated by the known receptor complex IL-20RA/IL-10RB. In fact, we identified EphA3 as a novel functionally interacting receptor for IL-26 in TNBC. Human EphA3 is a 110 kDa protein consisting of 983 amino acids and belongs to the ephrin receptor subfamily of the RTK family. EphA3 is well conserved between human and mouse with ~96% homologies and 88% similarities^48^, allowing human IL-26 to bind to both human and murine EphA3 on TNBC cells. Mutations of EphA3 were identified as candidate cancer risk genes in breast cancer, colon cancer, lung cancer, glioblastoma, melanoma, and pancreatic carcinoma^33,49–52^. The roles of the EPH family in cancer are controversial. Overexpression of EPH family can promote and inhibit tumor progression, even in the same tumor type^33,53,54^. Previous reports showed that a preferential binding ligand for EphA3 is ephrin-A5^33^. Many cancer cell lines and normal endothelial cells release A-type ephrins from their cell surface^55^. Membrane-bound ephrin-A5 ligand-induced EphA3 clustering and its tyrosine phosphorylation, followed by activation of GTPase or ERK and dephosphorylation of AKT, resulting in cytoskeletal re-organization, cell retraction, and differentiation^56^. A-type ephrins, particularly ephrin A1, A4, and A5, can be released from the cell surface through metalloproteases, phospholipases, or through alternative splicing, which removes the exon encoding the site of GPI anchor attachment^55,57,58^. Binding of unbound, soluble ephrin-A5 to EphA3 resulted in dephosphorylation of EphA3 by recruitment of protein tyrosine phosphatase and auto-inhibition of its RTK domain, leading to cell growth and proliferation^56,59,60^. However, our present study showed that the addition of exogenous ephrin-A5 has little impact on the effects of gefitinib in TNBC cells. It is possible that the EphA3 antibody may reduce the effect of IL-26 on gefitinib by preventing access to the IL-26 binding site, not by blocking ephrin-A5 ligand binding. These results suggest that the blockade of IL-26 overcomes EGFR-TKI resistance in TNBC. In the current study, exogenous IL-26 induced dephosphorylation of EphA3 in TNBC, followed by phosphorylation of AKT and JNK leading to suppression of ER stress signaling, resulting in enhanced tumor growth of EGFR-TKI-resistant TNBC. Despite the EphA3-related molecular alterations associated with IL-26 alone, treatment with IL-26 alone did not alter TNBC growth; rather, the effect of IL-26 on TNBC tumor growth was seen in the setting of EGFR-TKI exposure with an increased ER stress response. Our present study did not demonstrate direct IL-26 and EphA3 binding. IL-26 has been reported to be a cationic and amphipathic cytokine, resembling the structure of antimicrobial peptides^61^. Since polycationic proteins are known to bind with various molecules, it is, therefore, possible that IL-26 forms a large complex that includes EphA3 to affect downstream signaling.

In conclusion, we showed that IL-26 activates an EGFR-TKI-associated bypass pathway in TNBC and that EphA3 is a putative novel functional receptor for IL-26 in TNBC, and binding of IL-26 inhibits ER stress signaling via phosphorylation of AKT and JNK downstream of EphA3, leading to cell growth. Blockade of IL-26 overcomes EGFR-TKI resistance in TNBC. These findings reveal the critical importance of IL-26 on EGFR-TKI resistance in TNBC, and serve as the basis for a novel therapeutic strategy in TNBC, as well as potentially other EGFR-TKI resistant cancers such as non-small cell lung cancer or colorectal cancer, with the combination of anti-IL-26 mAb and anti-EGFR agents. In addition, this therapeutic approach may be considered for other medical conditions involving IL-26 and EphA3, such as pulmonary fibrosis^13,14,40,62,63^. By clarifying the mechanisms involved in IL-26-mediated EGFR-TKI resistance in TNBC, our work solves the paradox involving the cross-species difference between murine models and human subjects observed with EGFR-TKI treatment of TNBC.

## Materials and methods

### Cells, reagents, and antibodies

The mouse breast carcinoma cell line E0771 was purchased from CH3 Biosystems (Amherst, NY). The human breast adenocarcinoma cell lines HCC70, MDA-MB468, and MDA-MB231 were obtained from ATCC. All cell lines had been tested for mycoplasma. For cell stimulation, a recombinant human IL-26 dimer was purchased from R&D Systems (Minneapolis, MN). Recombinant human IL-26 dimer was labeled with Alexa Fluor 488 utilizing protein labeling kit (ThermoFisher Scientific, Waltham, MA) and used for immunofluorescence staining. Recombinant human and mouse EphA3 Fc Chimera Protein were purchased from R&D Systems. EGFR-TKIs and inhibitors used in this study are shown in Supplementary Table S1. siRNAs against EphA3 were purchased from ThermoFisher Scientific (sequences are shown in Supplementary Table S2), and negative control siRNA (oligonucleotide sequences are not disclosed) was purchased from Qiagen (Hilden, Germany). FuGENE HD Transfection Reagent (Promega, Madison, WI) was used to transfect plasmid pCMV6-ENTRY EPHA3 or pCMV6-ENTRY control (ORIGENE, Rockville, MD) into MDA-MB231 cells. Antibodies used in this study are listed in Supplementary Table S3.

### Mice

hIL-26Tg mice were kindly provided by Dr. Thomas Aune and were interbred with C57BL/6 mice in the animal facility in Juntendo University. All mice used in this study were kept under the specific pathogen-free facility in microisolator cages. Female hIL-26Tg mice and littermate control mice at 8–12 weeks of age were used.

### MTT assay

Human and mouse breast carcinoma cells (1 × 10^4^) were incubated in RPMI 1640 containing 10% FCS on 96-well flat-bottom plates (Corning, Tewksbury, MA) for 24 h at 37 °C, and then stimulated with IL-26 in the presence or absence of EGFR-TKI (Gefitinib or Erlotinib), signal inhibitors and neutralizing antibodies for 48 h. Tetrazolium monosodium salt (WST-8) was added to each well at a concentration of 1/10 volume for the last 1 h, and the absorbance at 450 nm/595 nm was measured using a Microplate Reader (Bio-Rad, Hercules, CA) and data were analyzed with Microplate Manager 6 software (Bio-Rad).

### Western blotting

To analyze phosphorylation of AKT, ERK, p38, JNK, STAT3, EphA3, PERK, eIF2α and IRE1, E0771, and HCC70 cells were stimulated with IL-26 (30 ng/ml) in the presence or absence of Gefitinib and anti-EphA3 pAb (10 μg/ml) in 100-mm dish for the indicated periods. After stimulation, cells were lysed in RIPA buffer supplemented with 2% protease inhibitor mixture (Sigma-Aldrich, Saint. Louis, MO) and 1 × PhosSTOP (Roche Diagnostics, Tokyo, Japan), and lysates were resolved by SDS-PAGE in reducing condition (15 μg/lane) and immunoblotted using anti-phosphorylated AKT, ERK, p38, JNK, STAT3, EphA3, PERK, eIF2α, and IRE1 antibodies recognizing both human and murine antigens. For reprobing, the membranes were submerged in a stripping buffer. After a stripping procedure, the membranes were reprobed with anti-pan AKT, ERK, p38, JNK, STAT3, EphA3, PERK, eIF2α, and IRE1 antibody recognizing both human and murine antigens. The images were taken using C-Digit blot scanner (MS Techno Systems Inc., Osaka, Japan).

### Flow cytometry

E0771, HCC70, and MDA-MB468 cells were washed in PBS containing 1% FBS and 0.1% sodium azide, and subsequently stained with fluorochrome-conjugated Abs (anti-IL-20RA and IL-10RB) for 30 min at 4 °C. E0771, HCC70, MDA-MB468, and MDA-MB231 cells were incubated with purified rabbit anti-human/mouse EphA3 pAb for 30 min at 4 °C, and subsequently stained with PE-conjugated donkey anti-rabbit IgG (BioLegend, San Diego, CA) for 25 min at 4 °C. Flow cytometry was performed on two-laser FACSCalibur (BD Biosciences, San Jose, CA), and data were analyzed with FlowJo software (BD Biosciences).

### Microarray analysis

E0771 cells were treated with exogenous IL-26 (30 ng/ml) for 6 or 24 h. Total RNA was isolated and subjected to DNA microarray analysis utilizing 3D-Gene mouse mRNA oligo chip (TORAY, Kamakura, Japan). A heat map of 960 genes differentially (fold change ≥ 2.0) expressed between vehicle- and IL-26-treated E0771 cells was constructed by hierarchical cluster analysis using cluster 3.0 software, with results displayed with the TreeView program. HCC70 cells were treated with exogenous IL-26 (30 ng/ml) in the presence or absence of Gefitinib (40 μM) for 3 h. Total RNA was subjected to DNA microarray analysis with 3D-Gene human mRNA oligo chip. A heat map of 943 genes differentially (fold change ≥ 2.0) expressed between vehicle-, IL-26-, gefitinib plus vehicle-, and gefitinib plus IL-26-treated HCC70 cells were constructed and displayed as described above. The fraction of gene categories identified by the microarray Gene Ontology (GO) enrichment analysis is shown as GO terms in descending order of correlation coefficient values. The data discussed in this publication have been deposited in NCBI’s Gene Expression Omnibus and are accessible through GEO Series accession number GEO accession: GSE147804 (E0771), GSE171641(HCC70).

### In vivo evaluation of IL-26 in a TNBC transplantation model

E0771 cells (5 × 10^5^) with 50% Matrigel were injected subcutaneously into the flank of hIL-26Tg or control mice. Tumor measurements were made using calipers and volumes were calculated using the formula (*v* = width × width × (length/2)). After 10 days from E0771 cells injection, hIL-26Tg or control mice were randomly selected for treatment with vehicle control, gefitinib or each antibody (*n* = 8; calculated by power analysis based on our pilot studies). Gefitinib was suspended in H_2_O containing 1% Tween 80 for administration by oral gavage (50 mg/kg). Oral gavage was administered once a day 5 times a week for the duration of the study. H_2_O containing 1% Tween 80 and no Gefitinib was used as a control. For mAb treatment, anti-IL-26 mAb (clone 69-10) developed in our laboratory^42^ or mouse IgG1 isotype control was diluted in sterile PBS at 1 mg/ml and 200 μl (200 μg) was injected intraperitoneally once a day twice a week from 10 days after E0771 cells injection. Anti-EphA3 pAb (R&D Systems) or goat IgG isotype control was diluted in sterile PBS at 500 μg/ml and 200 μl (100 μg) was injected intraperitoneally once a day twice a week from 10 days after E0771 cells injection. Mice were sacrificed when subcutaneous tumor size reached 1600 mm^3^. No blinding was involved in animal studies.

### Immunofluorescence analysis

E0771 and HCC70 cells (1 × 10^5^) were incubated in RPMI 1640 containing 10% FCS on Lab-Tek chamber slide (ThermoFisher Scientific) for 24 h. After incubation, cells were stimulated with Alexa Fluor 488-labeled IL-26 (30 ng/ml) in the presence or absence of recombinant human and mouse EphA3-Ig for 1 h. HCC70 cells were stimulated with IL-26 (30 ng/ml) in the presence or absence of Gefitinib (40 μM), signal inhibitors, and neutralizing antibodies for 24 h. Subcutaneous tumor samples obtained from mice were fixed in 4% paraformaldehyde (ThermoFisher Scientific), embedded in OCT compound (Tissue-Tek, Sakura Finetek, Tokyo, Japan). Cells and slides were immunostained with each antibody and observed utilizing Zeiss inverted microscope and Apotome.2. program (Carl Zeiss, Oberkochen, Germany). Fluorescence intensity was quantitated using Image-J software (NIH). Images were captured using objectives of ×100–×400.

### In situ proximity ligation assay (PLA)

HCC70 cells (1 × 10^5^) were incubated in RPMI 1640 containing 10% FCS on Lab-Tek chamber slide (ThermoFisher Scientific) for 24 h. After incubation, cells were stimulated with recombinant human IL-26 (30 ng/ml) for 1 h. Following stimulation, cells were fixed with 4% paraformaldehyde in PBS for 15 min at RT. The coverslips were incubated with goat anti-human IL-26 pAb (R&D systems) and mouse anti-human EphA3 mAb (Santa Cruz Biotechnology, Santa Cruz, CA), and subjected to in situ PLA using the Duolink Detection kit (ThermoFisher Scientific) according to the manufacturer’s instructions. The coverslips were incubated with PLA minus and PLA plus probes (anti‐goat PLUS and anti‐mouse MINUS diluted in the antibodies diluent provided with the kit) for 1 h at 37 °C in a humidity chamber. The coverslips were washed and processed for probe ligation, signal amplification, fluorescently labeled probe conjugation, and mounting. Cells were observed utilizing Zeiss inverted microscope and Apotome.2. program. Images were captured using objectives of ×400.

### JC-1 staining

Mitochondrial stability was assessed using a mitochondrial membrane potential (ΔΨ_m_) assay kit with JC-1 staining (Cayman Chemical, Ann Arbor, MI). HCC70 cells (1 × 10^4^) were stimulated with IL-26 (30 ng/ml) in the presence or absence of Gefitinib (40 μM), signal inhibitors, and neutralizing antibodies on flat-bottom plates 96-well (Corning) for 24 h. After stimulation, cells were stained for JC-1 Staining Solution. The ΔΨ_m_ was assessed using a fluorescence microscope (Nikon, Tokyo, Japan) at wavelengths of 530 and 590 nm, respectively. The ΔΨ_m_ depolarization displayed green fluorescence. Stained cells were quantitated using Image-J software (NIH). Images were captured using objectives of ×40.

### Quantitative real-time PCR (qRT-PCR)

E0771, HCC70, and MDA-MB468 cells were incubated in RPMI 1640 containing 10% FCS on 6-well plates (Corning) for 24 h, and then stimulated with IL-26 (30 ng/ml) in the presence or absence of Gefitinib for 3, 6, or 12 h. Cells were collected and total RNA was extracted. cDNA was synthesized using a PrimeScript II first strand cDNA synthesis kit (TaKaRa Bio, Shiga, Japan) with oligo (dT) primers. mRNA levels were measured by 7500 System SDS software (Applied Biosystems, Foster City, CA), being normalized to hypoxanthine phosphoribosyltransferase expression levels. Sequences of primers used in this study are shown in Supplementary Table S4.

### Tissue samples and immunohistochemistry

Pathological examinations were carried out by two experienced pathologists. On specimens subjected to IHC, ER status, and PgR status were determined semi-quantitatively and judged as positive when more than 1% of the nuclei of cancer cells showed staining. HER2 was determined as positive when more than 10% of tumor cells showed strong staining of the entire cell membrane. Triple-negative (ER/PgR-HER2-) status was classified when the samples were ER (<1%), PgR (<1%), and HER2 (<10%) by IHC staining. For an immunohistochemical double-staining of IL-26 and CD4, CD8, CD68 or CD163, antigen retrieval was performed by autoclaving in 10 mM citrate buffer (pH 6.0) for 10 min at 120 °C, and the sections were treated with 0.3% H_2_O_2_ in methanol for 10 min at RT to inactivate endogenous peroxidase. The sections were treated with rabbit anti-human IL-26 mAb, and subsequently treated with HRP-conjugated anti-rabbit Ig antibody (Vector Laboratories, Inc., Burlingame, CA) for 30 min at RT. The reaction was visualized with 3,3′-diaminobenzidine (DAB) (Dojindo Laboratories, Kumamoto, Japan). After DAB staining, the sections were washed in distilled water, then boiled in 10 mM citrate buffer (pH 6.0) for 5 min at 100 °C to remove antibodies. The sections were subsequently treated with mouse anti-human CD4 mAb, anti-CD8 mAb, anti-CD68 mAb, or anti-CD163 mAb, respectively at 4 °C overnight, and then treated with HRP-conjugated anti-mouse Ig antibody (Vector Laboratories, Inc). The reaction was visualized with Vector SG Reagent (Vector Laboratories, Inc), and the tissue sections were counterstained for the nucleus with hematoxylin. The optical images were taken using Zeiss inverted microscope and Axiovision 2.0 program. Histological studies were conducted in the Department of Breast Oncology of Juntendo University (Tokyo, Japan) after official approval of the Juntendo University School of Medicine Review Board was obtained (No: 17-252).

### Statistics

Data were analyzed by two-tailed Student *t*-test for two-group comparison or by one-way ANOVA test with Tukey’s for multiple comparison testing. Data are presented as mean ± S.D. of triplicate samples of the representative in vitro experiment, or mean ± S.E. of three independent in vivo experiments. Significance was analyzed using MS Excel (Microsoft) and values of *p* < 0.01 were considered significant and are indicated in the corresponding figures and figure legends.

## Supplementary information

Revised Supplementary Figures

Revised Legends to Supplementary Figures

## Data Availability

All data generated or analyzed during this study are included in this published article and its Supplementary Information files.
