# Supplementary material for: IL-26 mediates epidermal growth factor receptor-tyrosine kinase inhibitor resistance through endoplasmic reticulum stress signaling pathway in triple-negative breast cancer cells
Source: Cell Death Dis. 2021 May 21;12(6):520. doi: 10.1038/s41419-021-03787-5 (PMC8139965; doi:10.1038/s41419-021-03787-5)
Supplement: Supplementary file 2 — Revised Legends to Supplementary Figures [file 41419_2021_3787_MOESM2_ESM.pdf]

Legends to Supplementary Figures

Figure S1. Immunohistochemistry of HER2 and Luminal subtype clinical specimens.

HER2 (n=20) and Luminal (n=23) subtypes of breast carcinoma tissue specimens were stained with anti-human IL-26 mAb. All immunohistochemistry specimens were counterstained with hematoxylin. Representative images of 2 cases showing high IL-26 protein expression in HER2 and Luminal subtypes. IL-26 was detected in both tumor-infiltrating lymphocytes and tumor cells. Original magnification, 100x. Scale bar, 50 μm. Original magnification, 100x. Scale bar, 50 μm.

Figure S2. Exogenous IL-26 activates bypass pathway of EGFR-TKI in human TNBC in *in vitro* assays

A. Phase contrast microscopy of HCC70 cells (1×10^4) following 48 hr incubation with IL-26 or control vehicle in the presence or absence of gefitinib. Original magnification, 100x. Scale bar, 100 μm. Data shown are representative images of five independent experiments with similar results.

B. HCC70 cells were treated with the indicated dose of IL-26 in the presence or absence of gefitinib (40 μM) for 48 hr. IL-26 dose-dependently enhanced the proliferation of HCC70 cells in the presence of gefitinib. $*p<0.01$.

C. HCC70 cells were treated with IL-26 (30 ng/ml) in the presence of various doses of gefitinib (10, 20 or 40 μM). IL-26 significantly inhibited gefitinib-induced suppression of cell proliferation ($*p<0.01$), and the phenomenon was prominently observed with 40 μM gefitinib.

D. HCC70 cells were stimulated with IL-26 (30 ng/ml) for the indicated periods, and then submitted to Western blot analysis using anti-phosphorylated AKT, JNK, ERK, p38, and STAT3 antibodies, and reblotting with anti-pan AKT, JNK, ERK, p38, and STAT3 antibodies. Addition of IL-26 resulted in phosphorylation of AKT, JNK, ERK and p38 in HCC70 cells, but not in activation of STAT3.

E. HCC70 cells were stimulated with IL-26 (30 ng/ml) in the presence or absence of gefitinib (40 μM) for 15 min, and then submitted to Western blot analysis as described in D. IL-26 activated AKT and JNK signals in HCC70 cells even in the presence of gefitinib.

F. HCC70 cells were treated with IL-26 and/or gefitinib in the presence or absence of various concentrations of signal inhibitors (AKT inhibitor, JNK inhibitor or combination of AKT inhibitor and JNK inhibitor) for 48 hr. The dashed line is the standard value of gefitinib plus vehicle. Both AKT inhibitor and JNK inhibitor partially inhibited the proliferation of HCC70 cells in the absence of gefitinib. AKT inhibitor and JNK inhibitor hardly affected the proliferation of HCC70 cells treated with gefitinib alone. In contrast, combination of AKT inhibitor and JNK inhibitor almost completely reversed the inhibitory effect of IL-26 on gefitinib-induced suppression to the level similar with that of gefitinib alone. *$p<0.01$.

(B, C, F) Cell proliferation was assessed by MTT assay. Representative data of five (B) and three (C, F) independent experiments are shown as mean ± S.D. of triplicate samples, and similar results were obtained in each experiment.

(D, E) Data shown are representative of five independent experiments, and similar results were obtained in each experiment. Band intensity of phospho-proteins was normalized to the

appropriate pan proteins, and relative intensity compared with unstimulated cells is shown as mean ± SEM from five independent experiments. * p<0.01.

Figure S3. IL-26 overcomes the suppressive effect of gefitinib on MDA-MB468 cells.

A. Human TNBC MDA-MB468 cells were treated with the indicated dose of IL-26 in the presence or absence of gefitinib (20 μM) for 48 hr. IL-26 dose-dependently enhanced the proliferation of MDA-MB468 cells in the presence of gefitinib. *p<0.01.

B. MDA-MB468 cells were treated with IL-26 (30 ng/ml) in the presence of various doses of gefitinib (5, 10 or 20 μM). IL-26 significantly inhibited gefitinib-induced suppression of cell proliferation (*p<0.01).

(A, B) Cell proliferation was assessed by MTT assay. Representative data of three independent experiments are shown as mean ± S.D. of triplicate samples, and similar results were obtained in each experiment.

Figure S4. IL-26 overcomes the suppressive effect of erlotinib on TNBC cells.

(A, B, C) Mouse TNBC E0771 cells (A) and human TNBC HCC70 cells (B) and MDA-MB468 cells (C) were treated with IL-26 (30 ng/ml) in the presence of the indicated dose of erlotinib for 48 hr. MTT assays were conducted as described in Materials and Methods. IL-26 significantly inhibited erlotinib-induced suppression of cell proliferation (*p<0.01)

Representative data of three independent experiments are shown as mean ± S.D. of triplicate samples, and similar results were obtained in each experiment.

Figure S5. Colocalization of IL-26 and EphA3 was inhibited by addition of soluble EphA3. E0771 cells were treated with Alexa Fluor 488-labeled recombinant human IL-26 (rhIL-26-Alexa488 (green)) (30 ng/ml) in the presence of recombinant mouse EphA3-Ig (rmEphA3), human EphA3-Ig (rhEphA3) or control IgG (50 μg/ml, each) for 1 hr, followed by immunostaining with anti-mouse EphA3 pAb (red) and DAPI (blue). IL-26 was merged with cell surface EphA3 while both human and mouse soluble EphA3-Ig inhibited the interaction of IL-26 and cell surface EphA3. Original magnification 200x. Scale bar, 50 μm. Data shown are representative images of three independent experiments with similar results.

Figure S6. IL-20RA was not detected on human TNBC cell line MDA-MB468. MDA-MB468 cells were stained with the indicated Abs or isotype control, and analyzed by flow cytometry. Data are shown as histogram of IL-20RA (red lines), IL-10RB (blue lines) and EphA3 (red lines), and the gray area in each histogram shows isotype control data. Although expression of IL-10RB and EphA3 was detected on the cell surface of MDA-MB468 cells, IL-20RA, a major subunit of IL-26 receptor, was never observed on MDA-MB468 cells. Representative data of three independent experiments are shown, and similar results were obtained in each experiment.

Figure S7. EphA3 was expressed in TNBC cells of human clinical tissue samples A, B, C. TNBC tissue specimens were stained with H&E (A) and anti-human EphA3 pAb (B). All immunohistochemistry specimens were counterstained with hematoxylin. Representative images of 3 cases with IL-26-expressing cells and EphA3-expressing cells were shown. IL-26 was mainly detected in tumor-infiltrating lymphocytes, while expression of EphA3 was detected

in tumor cells. Original magnification, 40x. Scale bar, 100 μm. Similar results were obtained in 16 other TNBC samples.

Figure S8. EphA3 was not detected on human TNBC cell line MDA-MB231.

MDA-MB231 cells were stained with anti-EphA3 pAb or isotype control, and analyzed by flow cytometry. Data are shown as histogram of EphA3 (red lines), and the gray area in each histogram shows isotype control data. Although expression of EphA3 was not detected on the cell surface of MDA-MB231 cells and control plasmid-transfected cells, EphA3 was markedly expressed on EphA3-transfected MDA-MB231 cells. Representative data of three independent experiments are shown, and similar results were obtained in each experiment.

Figure S9. Expression level of DDIT3 was increased in gefitinib-stimulated human and mouse TNBC cells.

Human TNBC MDA-MB468 cells and mouse TNBC E0771 cells were treated with IL-26 (30 ng/ml) in the presence or absence of gefitinib (20 μM) for 6 hr. Total RNA was isolated, and cDNA was synthesized with oligo (dT) primers. mRNA expression levels were quantified by real-time RT-PCR, being normalized to hypoxanthine phosphoribosyltransferase 1 (HPRT1) expression levels. Although mRNA expression levels of DDIT3 in MDA-MB468 and E0771 cells were markedly increased following gefitinib treatment, IL-26 clearly decreased DDIT3 expression levels of gefitinib-treated MDA-MB468 and E0771. *p<0.01.

Representative data of three independent experiments are shown as mean ± S.D. of triplicate samples, and similar results were obtained in each experiment.

Figure S10. Expression levels of inflammatory cytokines and chemokines were increased in gefitinib-stimulated human and mouse TNBC cells.

Cells were stimulated with IL-26 (30 ng/ml) in the presence or absence of gefitinib (40 μM for HCC70, and 20 μM for MDA-MB468 and E0771) for 6 hr. Total RNA was isolated, and cDNA was synthesized with oligo (dT) primers. mRNA expression levels of IL-6, IL-8, CXCL2 (MIP-2) and CXCL1 (KC) were quantified by real-time RT-PCR, being normalized to hypoxanthine phosphoribosyltransferase 1 (HPRT1) expression levels. Gefitinib treatment significantly enhanced mRNA expression levels of IL-6, IL-8 and CXCL2 in HCC70 and MDA-MB468, and IL-6, MIP-2 and KC in E0771. *$p<0.01$.

Representative data of three independent experiments are shown as mean ± S.D. of triplicate samples, and similar results were obtained in each experiment.

Figure S11. EphA3, phosphorylation of AKT and JNK play an important role in mitochondrial membrane potential of TNBC treated with IL-26 and gefitinib.

HCC70 cells were treated with IL-26 (30 ng/ml) and/or gefitinib (40 μM) in the presence of vehicle, signal inhibitors, anti-EphA3 pAb or anti-IL-26 mAb (clone 69-10) (50 μg/ml, each) for 24 hr, followed by JC-1 staining. Red fluorescence, sign of preserved mitochondrial membrane potential ($\Delta\Psi m$), was observed in vehicle- or IL-26-treated HCC70 cells in the absence of gefitinib, whereas green fluorescent signals, index of mitochondrial membrane depolarization were prominently observed in vehicle-treated HCC70 cells in the presence of gefitinib. Mitochondrial membrane depolarization was markedly reduced in IL-26-treated HCC70 cells

even in the presence of gefitinib. Both AKT inhibitor and JNK inhibitor partially increased mitochondrial membrane depolarization in IL-26-treated HCC70 cells in the presence of gefitinib. Combination of AKT inhibitor and JNK inhibitor almost completely reversed the inhibitory effect of IL-26 on gefitinib-induced mitochondrial membrane depolarization to the level similar with that of gefitinib alone. Both anti-EphA3 pAb and anti-IL-26 mAb reversed the effect of IL-26 on gefitinib-treated HCC70 cells, although the inhibitory effect of anti-IL-26 mAb was stronger than that of anti-EphA3 pAb. Stained cell clusters were quantified using Image-J software. *$p<0.01$.

Figure S12. Treatment with anti-IL-26 mAb alone or anti-EphA3 pAb alone had no effect on tumor growth in E0771 TNBC model.

A. E0771 cells (5×10^5) with Matrigel were injected subcutaneously into the flank of hIL-26Tg mice. Anti-IL-26 mAb (clone 69–10) or mouse IgG isotype control (each, 200 µg/dose) was injected intraperitoneally once a day twice a week from 10 days after E0771 inoculation. Administration of anti-IL-26 mAb alone did not suppress tumor growth in hIL-26Tg mice. n=6 mice for each group at each time point. mean ± SEM of each group, comparing values in hIL-26Tg mice treated with anti-IL-26 mAb to those in hIL-26Tg mice injected with isotype control mAb. NS denotes ‘not significant’.

B. E0771 cells (5×10^5) with Matrigel were injected subcutaneously into the flank of hIL-26Tg or control (WT) mice. Anti-EphA3 pAb or goat IgG isotype control (each, 100 µg/dose) was injected intraperitoneally once a day twice a week from 10 days after E0771 inoculation. Administration of anti-EphA3 pAb alone did not suppress tumor growth in hIL-26Tg mice or WT mice. n=5 mice for each group at each time point. mean ± SEM of each group, comparing values in hIL-26Tg

mice or WT mice treated with anti-EphA3 pAb to those in hIL-26Tg mice or WT mice injected with isotype control mAb. NS denotes ‘not significant’.

Figure S13. E0771 cells in TNBC models are exposed to IL-26 secreted by CD4+ T cells and macrophages infiltrating in the TME, evoking a resistance to EGFR-TKI therapy, associated with phosphorylation of AKT and JNK.

A, B, C, D, E, F, G. Immunofluorescence staining and H&E staining (A) of tumor specimens resected at 14 days after E0771 inoculation using anti-PECAM (green) (B), anti-CD4 (red) plus IL-26 (green) (C), anti-F4/80 (red) plus IL-26 (green) (D), Ly6G (green) (E), p-AKT (green) (F) or p-JNK (green) (G). PECAM-positive blood vessels (B) were markedly increased in hIL-26Tg mice treated with gefitinib (iv) than those in WT mice treated with gefitinib (iii) or hIL-26Tg mice treated with vehicle (ii). The number of $CD4^{+}$ T cells (C), $F4/80^{+}$ macrophages (D) and $Ly6G^{+}$ granulocytes (E) infiltrated in the TME of both gefitinib-treated WT mice (iii) and hIL-26Tg mice (iv) was significantly increased as compared with vehicle-treated WT mice (i) or hIL-26Tg mice (ii). In addition, the number of IL-26-expressing $CD4^{+}$ T cells (C) and $F4/80^{+}$ macrophages (D) in the TME of hIL-26Tg mice treated with gefitinib (iv) was increased as compared with those of hIL-26Tg mice treated with vehicle (ii), whereas IL-26 was never detected in WT mice treated with vehicle (i) nor gefitinib (iii). Phosphorylation of AKT (F) and JNK (G) in tumor cells of vehicle-treated hIL-26Tg mice (ii) was enhanced as compared with those of vehicle-treated WT mice (i). Although phosphorylation of AKT and JNK in tumor cells was strongly suppressed in gefitinib-treated WT mice (iii), phosphorylated AKT and JNK was clearly observed in hIL-26Tg mice in spite of gefitinib treatment (iv). Original magnification,

100x. Scale bar, 100 μm. Insets, higher magnification images of the boxed regions. Data shown are representative images of 8 mice in each group with similar results.
