# Supplementary material for: IL-26 mediates epidermal growth factor receptor-tyrosine kinase inhibitor resistance through endoplasmic reticulum stress signaling pathway in triple-negative breast cancer cells
Source: Cell Death Dis. 2021 May 21;12(6):520. doi: 10.1038/s41419-021-03787-5 (PMC8139965; doi:10.1038/s41419-021-03787-5)
Supplement: Supplementary file 1 — Revised Supplementary Figures [file 41419_2021_3787_MOESM1_ESM.pdf]

Supplementary Table 1

EGFR-TKI and inhibitor list

Product name	The primary target of the inhibition	* Conc	Company
Gefitinib (ZD1839)	EGFR	5 - 40 µM	Selleck Chemicals (Houston, TX, USA).
Erlotinib (CP358774)	EGFR	30, 50 µM	Selleck Chemicals
wortmannin	Akt	Low: 0.375, Mid: 0.75, High: 1.5 µM	Merck Millipore
SP600125	JNK	Low: 0.3, Mid: 1.0, High: 3.0 µM	Tocris Biosystems

Supplementary Table 2

EPhA3 siRNA

EPHA3 (GenBank accession number NM_005233)

Target gene		Oligo sequence
Sence 1 siRNA	Sense	CCAGUUAUGACAAUUAAGAtt
	Antisense	UCUUAAUUGUCAUAACUGGtg
Sence 2 siRNA	Sense	GCCUUCCAACGAAGUUAAUtt
	Antisense	AUUAACUUCGUUGGAAGGCtg

One hundred picomoles siRNA duplexes were transfected into $3x10^6$ HCC70 by using Lipofectamine RNAiMAX (Thermo Fisher Scientific) and OptiMEM medium according to the manufacturer’s instructions.

Supplementary Table 3

Antibody list

**MTT Assay, *in vivo* TNBC model**	
mouse IgG1,κ isotype control mAb (clone MG1-45)	BioLegend
goat IgG, isotype control pAb	R&D Systems
goat anti-mouse EphA3 pAb	R&D Systems
anti-human IL-26 mAbs (clone 2-2, 20-3, 31-4, 69-10)	Original (*mAbs* 2019)
goat anti-human IL-26 pAb	R&D Systems
**Immunofluorescence staining, In situ PLA**	
mouse anti-human EphA3 mAb (clone D-2)	Santa Cruz Biotechnology
goat anti-human IL-26 pAb	R&D Systems
Biotin-conjugated anti-human IL-26 mAb (clone 69-10)	Original (*mAbs* 2019)
rabbit anti-mouse CD31 pAb	abcam (Cambridge, MA)
rat anti-mouse CD4 mAb (clone GK1.5)	Thermo Fisher Scientific
rat anti-mouse Ly6g mAb (clone ab25377)	abcam
rat anti-mouse F4/80 mAb (clone CI:A3-1)	abcam
rabbit anti-phospho-Akt (Ser473) pAb	Cell Signaling Technology (Danvers, MA)
rabbit anti-phospho-JNK/SPAK (Thr183/Tyr185) mAb (clone 81E11)	Cell Signaling Technology
mouse anti- human/mouse DDIT3 mAb (clone 9C8)	abcam
rabbit anti-beta Tublin pAb- Loading Control	abcam
Alexa Fluor 488-conjugated donkey anti-rabbit IgG pAb	Thermo Fisher Scientific
Alexa Fluor 647-conjugated donkey anti-rat IgG pAb	Thermo Fisher Scientific
Alexa Fluor 488-conjugated streptavidin	Thermo Fisher Scientific
Alexa Fluor 647-conjugated goat anti-mouse IgG pAb	Thermo Fisher Scientific
Alexa Fluor 488-conjugated donkey anti-rat IgG pAb	Thermo Fisher Scientific
**Western blot analysis**	
rabbit anti-p38 mAb (clone D13E1)	Cell Signaling Technology
rabbit anti-phospho-p38 (Thr180/Tyr182) mAb (clone D3F9)	Cell Signaling Technology
rabbit anti-Akt pAb	Cell Signaling Technology
rabbit anti-phospho-Akt (Ser473) pAb	Cell Signaling Technology
rabbit anti-STAT3 pAb	Cell Signaling Technology
rabbit anti-phospho-STAT3 (Tyr705) pAb	Cell Signaling Technology
rabbit anti-JNK/SPAK pAb	Cell Signaling Technology
rabbit anti-phospho-JNK/SPAK (Thr183/Tyr185) mAb (clone 81E11)	Cell Signaling Technology
rabbit anti-ERK1/2 pAb	Cell Signaling Technology
rabbit anti-phospho-ERK1 (Thr202/Tyr204)/ERK2 (Thr185/Tyr187) pAb	Cell Signaling Technology
mouse anti-human EphA3 mAb (clone D-2)	Santa Cruz Biotechnology
mouse anti-human Eph receptor A3 (Y596) mAb (clone EPR3434(N))	abcam
rabbit anti-β-Actin mAb (clone 13E5)	Cell Signaling Technology
rabbit anti-PERK mAb (clone C33E10)	Cell Signaling Technology
rabbit anti-phospho-PERK (Ser555) pAb	Gene Tex (San Antonio, TX)
rabbit anti-eIF2α pAb	Cell Signaling Technology
rabbit anti-eIF2α (Ser51) mAb (clone D9G8)	Cell Signaling Technology
rabbit anti-IRE1 mAb (clone 14C10)	Cell Signaling Technology
rabbit anti-phospho-IRE1 (Ser724) pAb	Gene Tex
HRP-Linked sheep anti-mouse IgG	GE Healthcare
HRP-Linked Donkey anti-rabbit IgG	GE Healthcare
**Flow cytometry**	
rabbit anti-mouse IL-20RA pAb	LifeSpan BioSciences (Seattle, WA)
APC anti-mouse IL-10RB mAb (clone 547324)	R&D Systems
APC anti-human IL-20RA mAb (clone 173714)	R&D Systems
PE anti-human IL-10RB mAb (clone 90220)	R&D Systems
rabbit anti-EphA3 pAb	allomone labs (Jerusalem, Israel)
rabbit IgG, polyclonal - Isotype Control	BioLegend
PE mouse IgG1, Isotype Control (clone MOPC-21)	BioLegend
APC mouse IgG1, Isotype Control (clone MOPC-21)	BioLegend
PE anti-rabbit IgG	BioLegend
**Immunohistochemistry**	
rabbit anti-human IL-26 mAb (clone EPR22268-141)	abcam
rabbit anti-human EphA3 pAb	Gene Tex
HRP-Linked goat anti-rabbit IgG	abcam
mouse anti-human CD4 mAb (clone 4B12)	Nichirei Biosciences Inc (Tokyo, Japan)
mouse anti-human CD8 mAb (clone C8/144B)	Nichirei Biosciences Inc
mouse anti-human CD68 mAb (clone PG-M1)	DAKO (Carpinteria, CA)
mouse anti-human CD163 mAb (clone 10D6)	Novocastra Laboratories (Newcastle, United Kingdom)
secondary anti-rabbit Ig antibody (MP-7401)	Vector Laboratories, Inc
secondary anti-mouse Ig antibody (MP-7402)	Vector Laboratories, Inc

Supplementary Table 4

Primer list

Target gene		Oligo sequence
**Human**		
DDIT3	Sense	gctggaagcctggtatgag
	Antisense	ttgtgacctctgctggttctg
ATF3	Sense	ctaacctgacgccctttgtc
	Antisense	cgcctctttttcctttcatc
ATF4	Sense	tccagcgacaaggctaagg
	Antisense	ccaacagggcatccaagtc
ATF6	Sense	ttggaagcagcaaatgaga
	Antisense	ctgaccgaggagacgagac
IL-6	Sense	gccagagctgtgcagatgag
	Antisense	tcagcaggctggcatttg
IL-8	Sense	gtgcagagggttgtggagaagttt
	Antisense	cactggcatcttcactgattcttg
CXCL-2	Sense	ttcacctcaagaacatccaaa
	Antisense	caggaacagccaccaataag
HPRT	Sense	cagtcaacaggggacataaaag
	Antisense	cctgaccaaggaaagcaaag
**Mouse**		
DDIT3	Sense	atctcatccccaggaaacg
	Antisense	gtgcgtgtgacctctgttg
ATF3	Sense	ttgctaacctgacaccctttg
	Antisense	cgcctccttttcctctcatc
ATF4	Sense	gctatggatgatggcttgg
	Antisense	ttggactagaggggcaaaaag
IL-6	Sense	ttccatccagttgccttcttg
	Antisense	tttctcatttccacgatttcc
Mip-2	Sense	caccaaccaccaggctaca
	Antisense	cttcagggtcaaggcaaac
KC	Sense	ctgggattcacctcaagaaca
	Antisense	aagccagcgttcaccagac
HPRT	Sense	tgaagtactcattatagtcaagggca
	Antisense	ctggtgaaaaggacctctcg

Figure S1

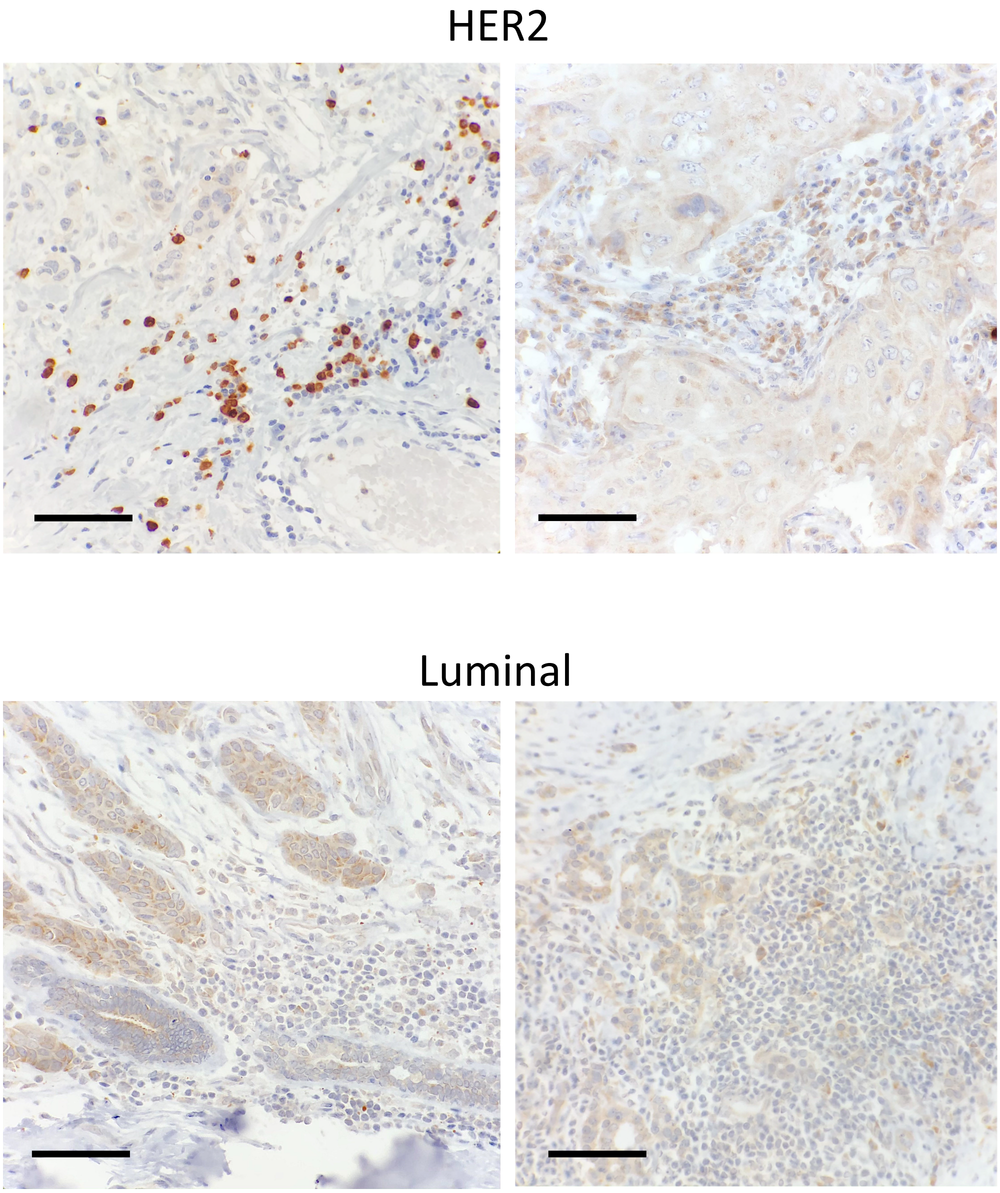

Figure S2

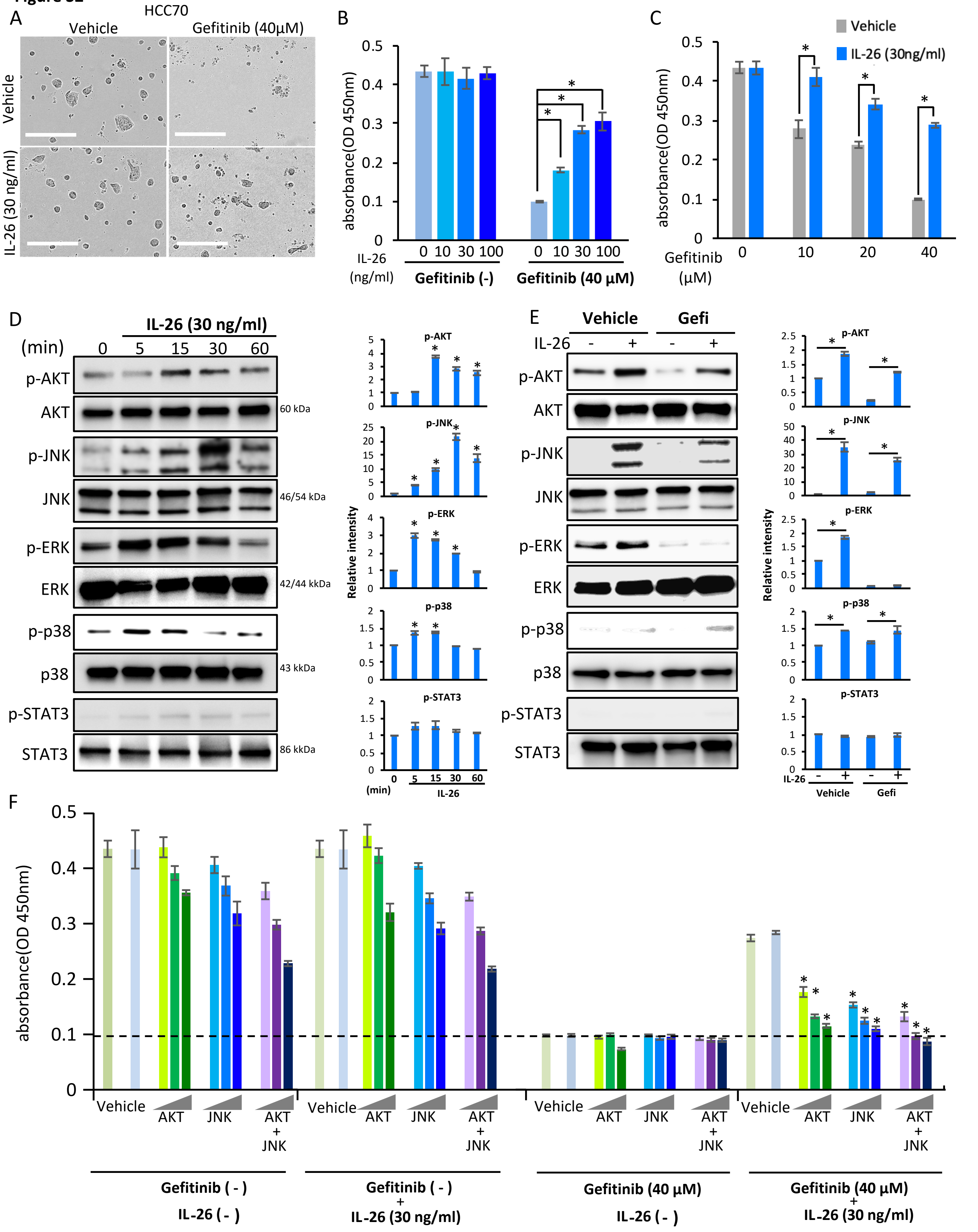

A
HCC70
Vehicle
Gefitinib (40μM)
Vehicle
IL-26 (30 ng/ml)
B
absorbance(OD 450nm)
IL-26 (ng/ml)
0 10 30 100
Gefitinib (-)
Gefitinib (40 μM)
C
Vehicle
IL-26 (30ng/ml)
absorbance(OD 450nm)
Gefitinib (μM)
0 10 20 40
D
IL-26 (30 ng/ml)
(min) 0 5 15 30 60
p-AKT
AKT
60 kDa
p-JNK
JNK
46/54 kDa
p-ERK
ERK
42/44 kkDa
p-p38
p38
43 kkDa
p-STAT3
STAT3
86 kkDa
Relative intensity
(min)
IL-26
E
Vehicle
Gefi
IL-26 - + - +
p-AKT
AKT
p-JNK
JNK
p-ERK
ERK
p-p38
p38
p-STAT3
STAT3
Relative intensity
F
absorbance(OD 450nm)
Vehicle
AKT
JNK
AKT + JNK
Gefitinib (-)
IL-26 (-)
Gefitinib (-) + IL-26 (30 ng/ml)
Gefitinib (40 μM)
IL-26 (-)
Gefitinib (40 μM) + IL-26 (30 ng/ml)

Figure S3

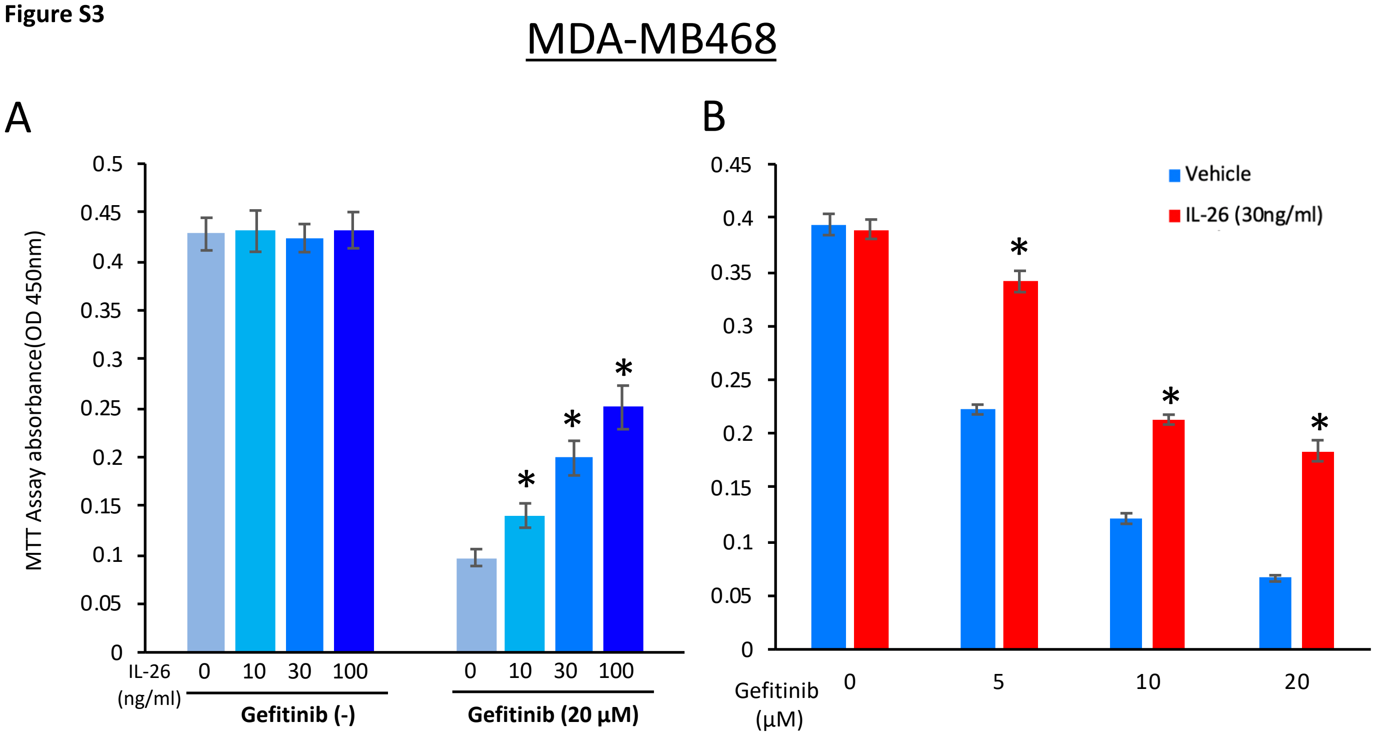
MDA-MB468
A
MTT Assay absorbance(OD 450nm)
0.5
0.45
0.4
0.35
0.3
0.25
0.2
0.15
0.1
0.05
0
IL-26
(ng/ml)
0 10 30 100
Gefitinib (-)
0 10 30 100
Gefitinib (20 µM)
*
*
*
B
0.45
0.4
0.35
0.3
0.25
0.2
0.15
0.1
0.05
0
Vehicle
IL-26 (30ng/ml)
Gefitinib
(µM)
0 5 10 20
*
*
*

Figure S4

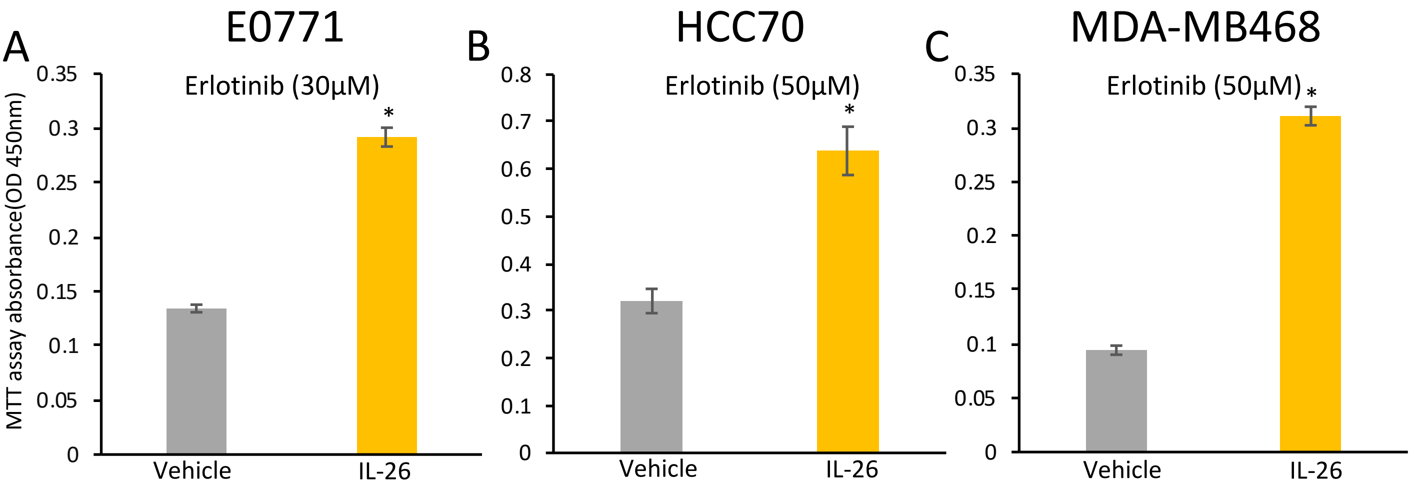
A
E0771
Erlotinib (30µM)
MTT assay absorbance(OD 450nm)
0.35
0.3
0.25
0.2
0.15
0.1
0.05
0
*
Vehicle
IL-26
B
HCC70
Erlotinib (50µM)
0.8
0.7
0.6
0.5
0.4
0.3
0.2
0.1
0
*
Vehicle
IL-26
C
MDA-MB468
Erlotinib (50µM)
0.35
0.3
0.25
0.2
0.15
0.1
0.05
0
*
Vehicle
IL-26

Figure S5

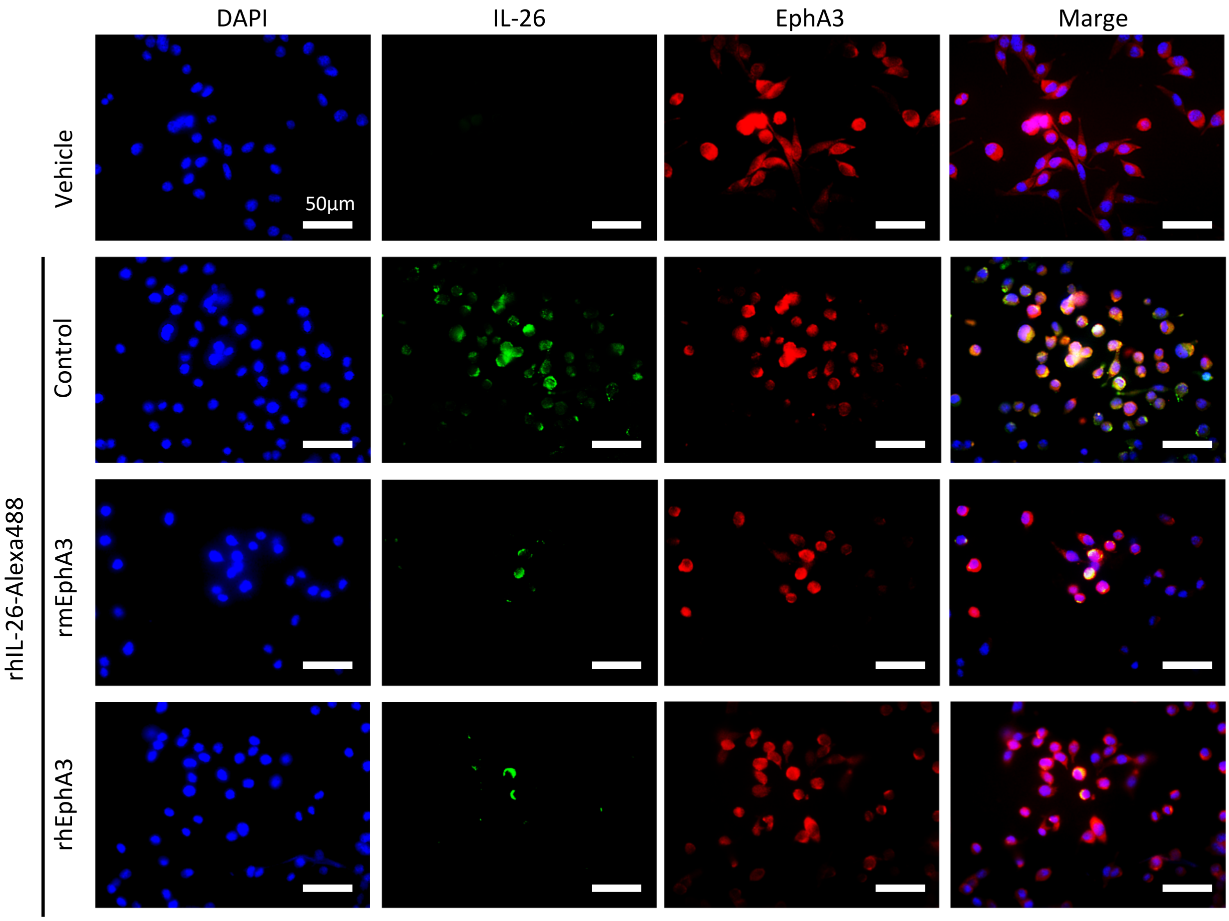
DAPI
IL-26
EphA3
Marge
Vehicle
50µm
Control
rmEphA3
rhEphA3
rhIL-26-Alexa488

Figure S6

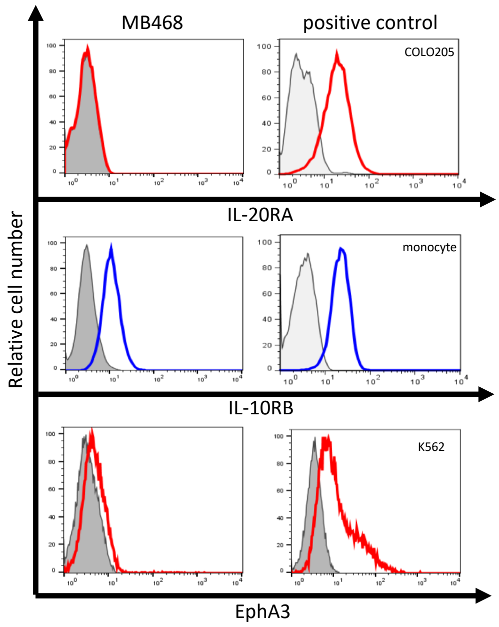
MB468
positive control
COLO205
IL-20RA
monocyte
IL-10RB
K562
EphA3
Relative cell number

Figure S7

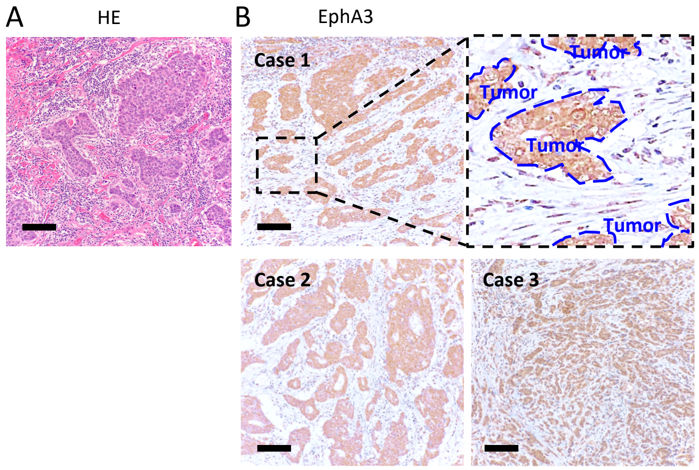

Figure S8

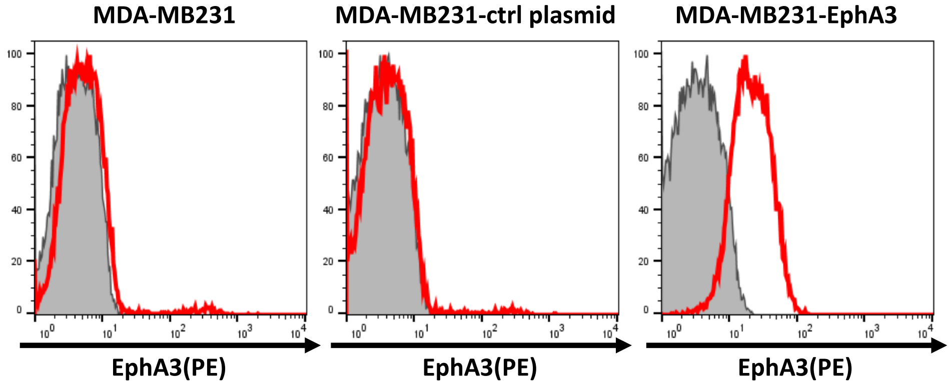
MDA-MB231
MDA-MB231-ctrl plasmid
MDA-MB231-EphA3
EphA3(PE)
EphA3(PE)
EphA3(PE)

Figure S9

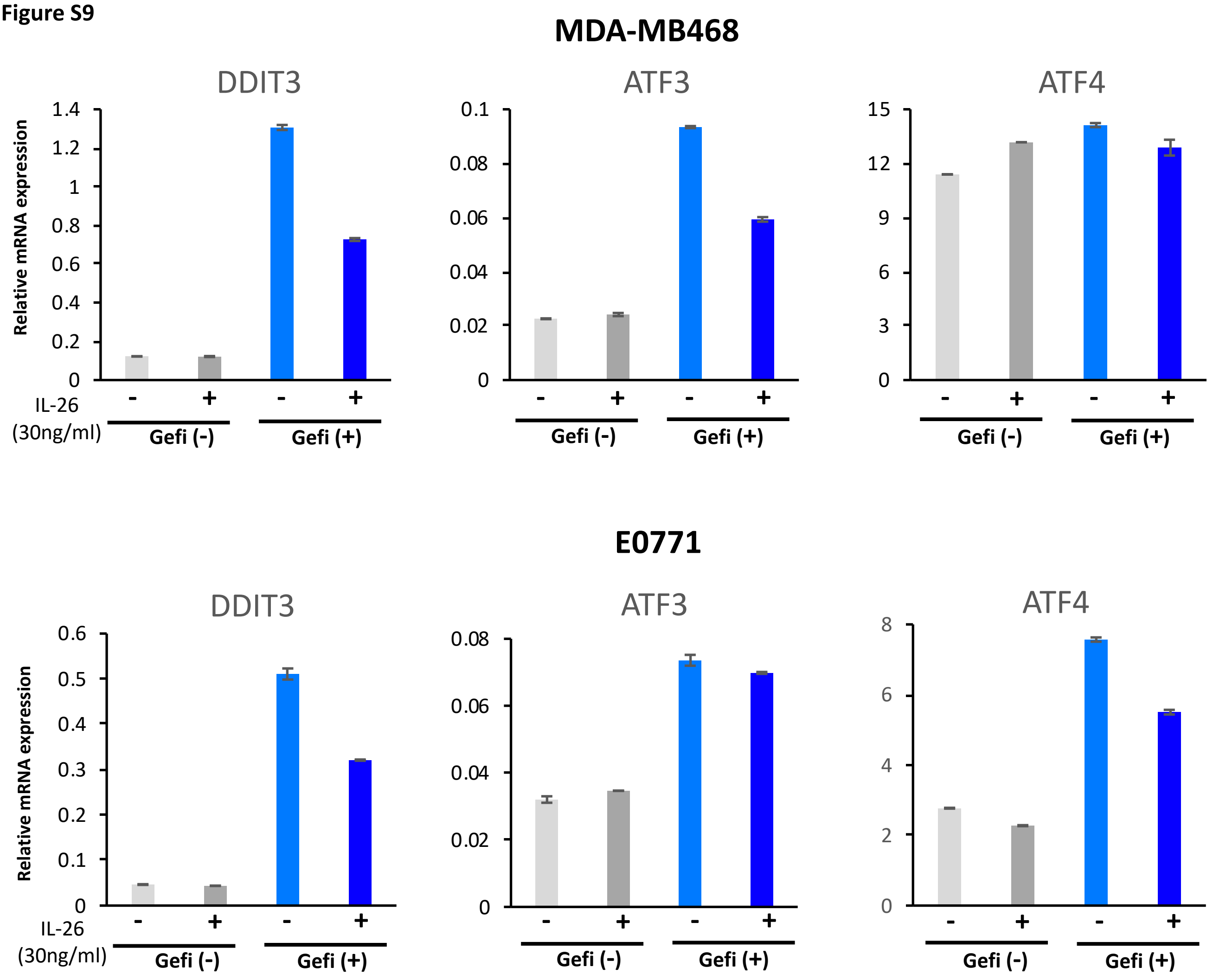
MDA-MB468
DDIT3
ATF3
ATF4
Relative mRNA expression
1.4
1.2
1
0.8
0.6
0.4
0.2
0
0.1
0.08
0.06
0.04
0.02
15
12
9
6
3
IL-26
(30ng/ml)
-
+
Gefi (-)
Gefi (+)
E0771
DDIT3
ATF3
ATF4
Relative mRNA expression
0.6
0.5
0.4
0.3
0.2
0.1
0
0.08
0.06
0.04
0.02
8
6
4
2
IL-26
(30ng/ml)
Gefi (-)
Gefi (+)

Figure S10

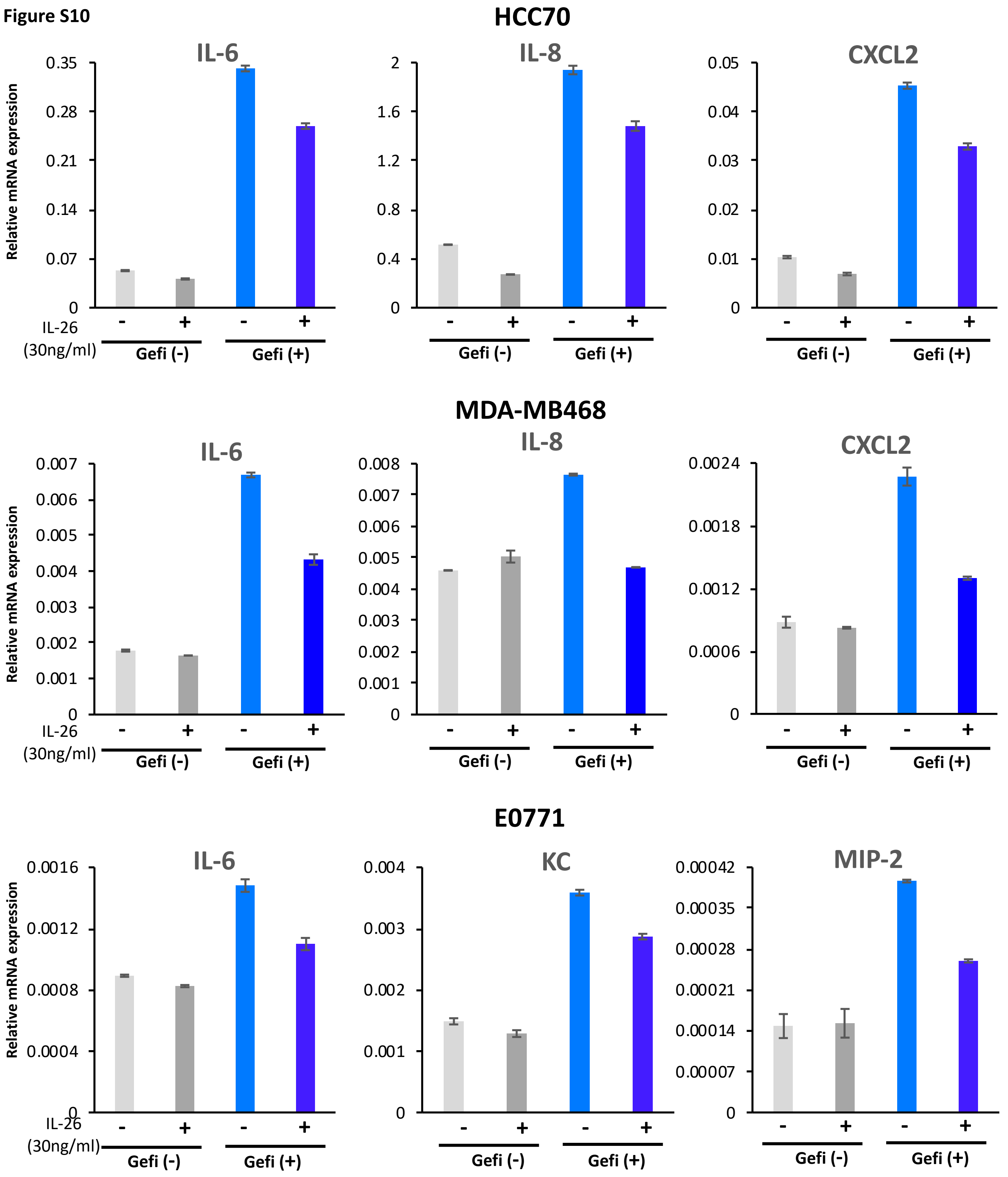
HCC70
IL-6
IL-8
CXCL2
Relative mRNA expression
0.35
0.28
0.21
0.14
0.07
0
2
1.6
1.2
0.8
0.4
0.05
0.04
0.03
0.02
0.01
IL-26
(30ng/ml)
- + - +
Gefi (-)
Gefi (+)
MDA-MB468
IL-6
IL-8
CXCL2
0.007
0.006
0.005
0.004
0.003
0.002
0.001
0.008
0.0024
0.0018
0.0012
0.0006
E0771
IL-6
KC
MIP-2
0.0016
0.0012
0.0008
0.0004
0.004
0.003
0.002
0.001
0.00042
0.00035
0.00028
0.00021
0.00014
0.00007

Figure S11

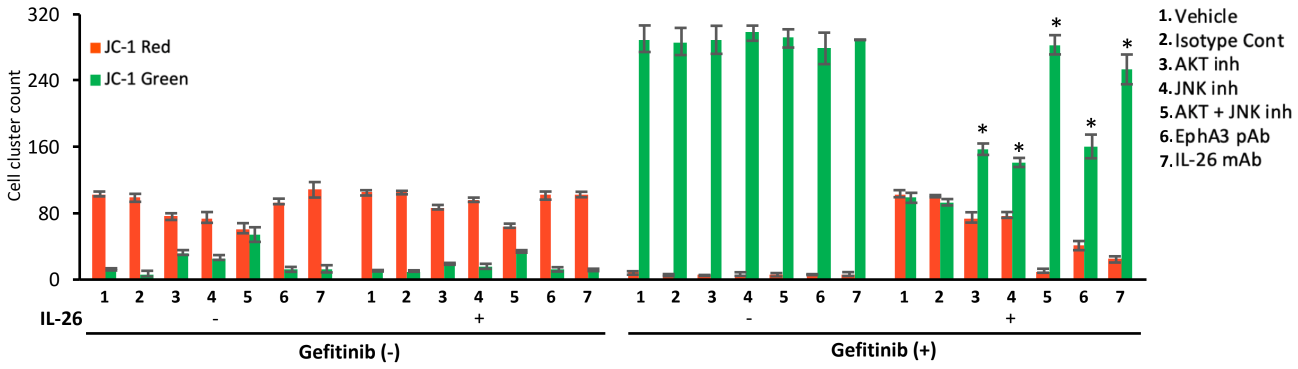

320
240
160
80
0
JC-1 Red
JC-1 Green
Cell cluster count
*
*
*
*
*
*
1 2 3 4 5 6 7 1 2 3 4 5 6 7 1 2 3 4 5 6 7 1 2 3 4 5 6 7
IL-26
-
+
-
+
Gefitinib (-)
Gefitinib (+)
1. Vehicle
2. Isotype Cont
3. AKT inh
4. JNK inh
5. AKT + JNK inh
6. EphA3 pAb
7. IL-26 mAb

Figure S12

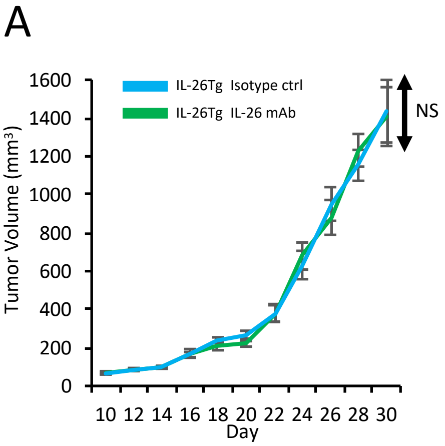
A
IL-26Tg Isotype ctrl
IL-26Tg IL-26 mAb
NS
Tumor Volume (mm³)
1600
1400
1200
1000
800
600
400
200
0
10 12 14 16 18 20 22 24 26 28 30
Day

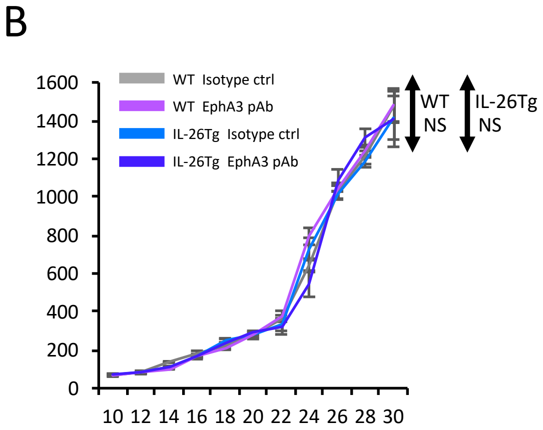
B
WT Isotype ctrl
WT EphA3 pAb
IL-26Tg Isotype ctrl
IL-26Tg EphA3 pAb
WT
NS
IL-26Tg
NS
1600
1400
1200
1000
800
600
400
200
0
10 12 14 16 18 20 22 24 26 28 30

Figure S13

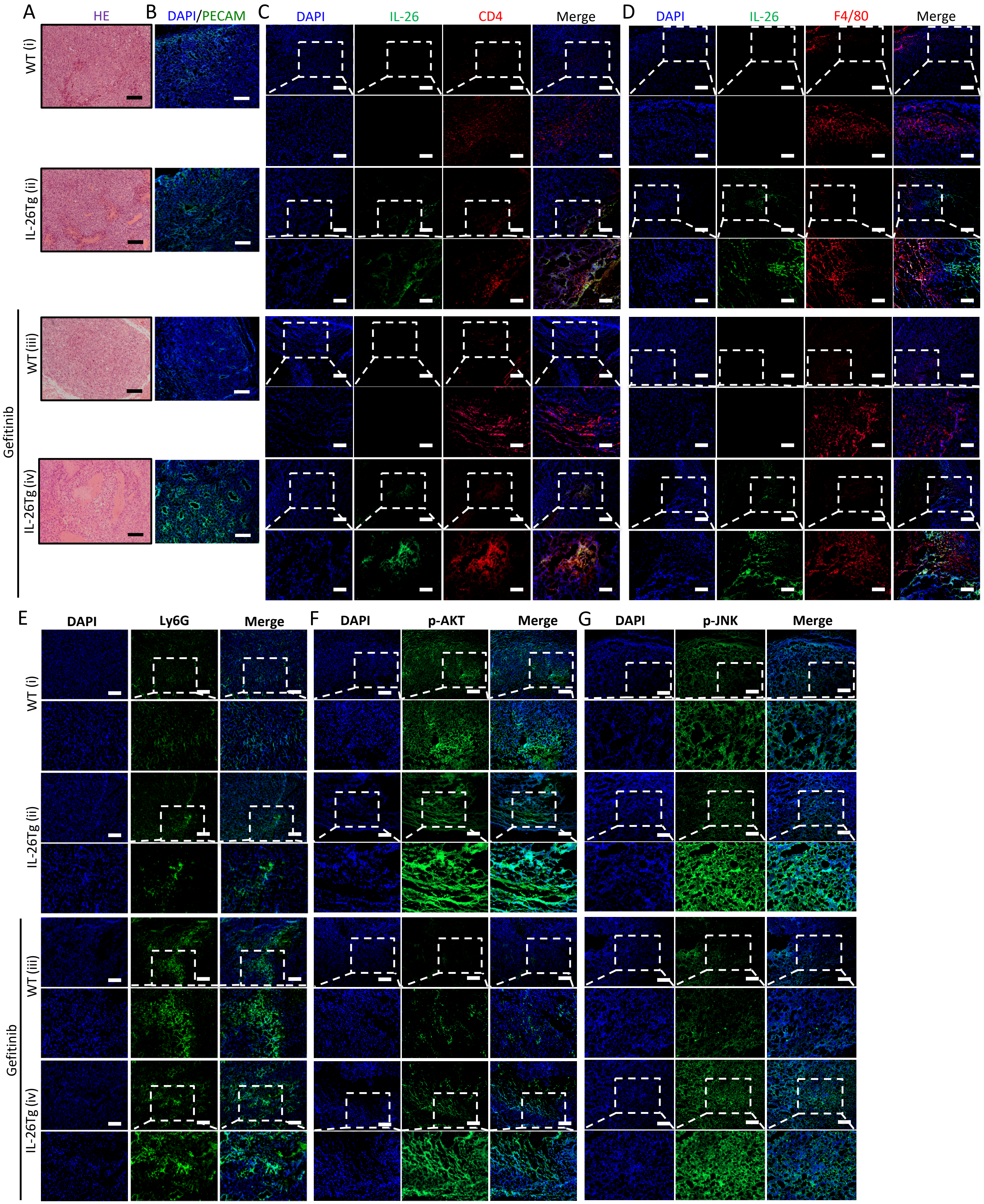

A
HE
B
DAPI/PECAM
C
DAPI
IL-26
CD4
Merge
D
DAPI
IL-26
F4/80
Merge
WT (i)
IL-26Tg (ii)
WT (iii)
IL-26Tg (iv)
Gefitinib
E
DAPI
Ly6G
Merge
F
DAPI
p-AKT
Merge
G
DAPI
p-JNK
Merge
WT (i)
IL-26Tg (ii)
WT (iii)
IL-26Tg (iv)
Gefitinib
